# Hydrazine Derivatives as C-Centered Radical Precursors for C-C Bond Formation Reactions

**DOI:** 10.3390/molecules31010067

**Published:** 2025-12-24

**Authors:** Elena R. Lopat’eva, Igor B. Krylov, Alexander O. Terent’ev

**Affiliations:** N. D. Zelinsky Institute of Organic Chemistry, Russian Academy of Sciences, 47 Leninsky Prospekt, 119991 Moscow, Russia; elena.lopatyeva@gmail.com

**Keywords:** acyl hydrazide, arylhydrazine, hydrazine, carbazate, radical chemistry, C-C coupling

## Abstract

Organic monosubstituted hydrazine derivatives (Ar-NHNH_2_, RC(O)-NHNH_2_, Alkyl-NHNH_2_) are synthetically available, atom-efficient and stable sources of C-centered radicals upon oxidation with extrusion of the energetically favorable N_2_ molecule. This review summarizes the synthetic application of monosubstituted hydrazine derivatives (arylhydrazines, carbazates, acylhydrazides, hydrazine carboxamides and alkylhydrazines) in free-radical C-C bond-forming reactions. The main application directions in this field are (a) alkene difunctionalization, (b) cascade cyclization initiated by the addition of hydrazine-derived C-centered radicals to acrylamides and isonitriles, and (c) CH-functionalization of (hetero)arenes via C-centered radical addition followed by oxidative dehydrogenation (re-aromatization).

## 1. Introduction

The first examples of the carbon-centered radical generation from hydrazine derivatives started emerging at the end of the 20th century [1]. Initially, aryl hydrazides were recognized as convenient radical sources in the reaction of arylhydrazines with different copper(II) salts. The applicability of this finding was shown by the addition of thus generated aryl radicals to alkenes [1]. The first well-documented example of the use of acylhydrazines as sources of acyl radicals for various organic transformations dates back to 2002 [2]. This article showed the utility of acyl hydrazides in intramolecular cyclization reactions and in the trapping of acyl radicals with TEMPO.

It should be noted that apart from synthetic applications, acyl hydrazides represent an important compound class in drug design. The most well-known hydrazide drug—isoniazid—was synthesized in the early 20th century and still used for the treatment of tuberculosis. It is interesting that the mechanism of its action is related to the generation of reactive acyl radicals from it under physiological conditions [3,4,5,6].

**Figure 1:**
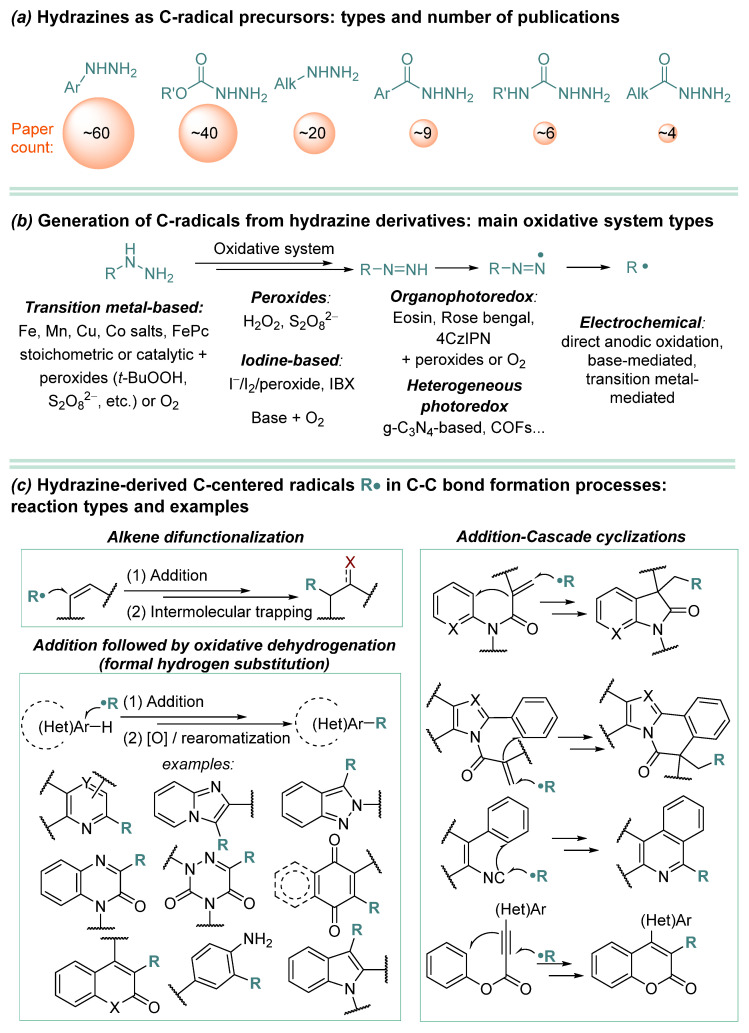
The scope of the present review.

Currently, hydrazine derivatives (HD) constitute a privileged class of C-centered radical precursors for diverse synthetic applications, for example, alkene difunctionalization, cascade cyclizations and (hetero)arenes CH-functionalization. HD are distinguished by low cost and commercial availability, straightforward synthesis, air and moisture stability, bench stability, the absence of greenhouse gas emissions upon radical generation (in contrast to decarboxylation processes) and low waste production (in contrast to reagents using larger leaving groups compared to the N_2_ molecule). Main hydrazine classes used as carbon-centered radical precursors are arylhydrazines and carbazates (Figure 1a), whereas alkylhydrazines, acylhydrazines and hydrazine carboxamides were used less frequently. HD oxidation proceeds sequentially via the formation of intermediate *N*-centered radicals and diazene intermediate (Figure 1b). While there are examples of oxidative coupling where hydrazine moiety is retained [7,8], such processes are rare compared to oxidation with loss of N_2_ molecule and formation of C-centered radicals.

Diazene intermediates are not so well studied. Phenyldiazene (PhN=NH) was synthesized in the 1960s by decarboxylation of PhN=NCO_2_^−^ anion [9,10] and was characterized by fast bimolecular self-decay to benzene and N_2_ precluding its isolation in pure state, as well as instability in the presence of molecular oxygen, bases and acids [10]. Later, aryldiazenes were trapped by furane derivatives, presumably by [2+4] cycloaddition [11,12]. Acyldiazenes are known as highly reactive intermediates, which can undergo further oxidation to acyl radicals or undergo nucleophilic attack on carbonyl carbon atom serving as electrophilic acylating agents [2,13,14].

So, the interception of diazene intermediates in RNHNH_2_ oxidation is rare, the dominant and highly exergonic route involves further oxidation with the extrusion of N_2_ molecule and the formation of a carbon-centered radical. Furthermore, if the corresponding radical is either acyl or carboxyalkyl radical, it can undergo decarbonylation [15] or decarboxylation [15,16,17], respectively, to yield a new carbon-centered radical species. The scope of oxidative systems for generation of C-radicals from hydrazines is extremely diverse, including transition metal-based systems, peroxides, iodine compounds, photoredox catalysts, electrochemical approaches, or just molecular oxygen under basic conditions (Figure 1b).

Given the extensive recent coverage of sulfonyl hydrazides in the recent literature [18,19,20,21,22,23,24,25], mainly as precursors of sulphonyl radicals, this review will focus specifically on the radical reactivities of acyl hydrazides, aryl and alkylhydrazines, carbazates and hydrazine carboxamides. The ability of such synthetically available hydrazine reagents to easily generate carbon-centered radicals under action of oxidants has established them as powerful tools in synthetic radical chemistry. Despite their broad utility and recognition within the field, a comprehensive review that systematically categorizes these diverse reaction pathways, elucidates the underlying mechanisms and provides predictive guidelines for their application is currently lacking. Selected electrochemical and photochemical radical coupling reactions of hydrazines were highlighted in a recent review [26]. Generation of aryl radicals from arylhydrazines was considered in a general review of aryl radical generation methods by redox processes [27], in reviews of (hetero)arenes radical arylation [28,29,30], reviews of photo- [31] and electrochemical [31,32] aryl radical generation, and in a review of synthetic applications of arylhydrazines in photoinduced transformations [33]. However, arylhydrazines were practically not discussed in a recent general review of contemporary methods for generation of aryl radicals [34], which indicates that this topic is not highlighted sufficiently in the literature despite numerous research advances.

The chemistry of organic hydrazine derivatives as carbon radical precursors is too rich to be placed into one review. In some reaction types, C-centered radical formation is discussed, but not accepted generally, as other reaction pathways are possible. For example, hydrazines are widely used as reagents for transition-metal-catalyzed cross-coupling, where carbon-centered radicals are considered as plausible intermediates. This review does not cover reactions, where C-radicals derived from hydrazines form metalloorganic species (in reactions, catalyzed by Au [35], Cu [36,37], Ni [38] and Pd [39,40,41,42,43,44]) without directly attacking the substrate.

Since the methods of generating C-radicals from various hydrazine derivatives are similar, published results are arranged in the present review according to the reaction types determined by the nature of C-centered radical acceptors. Main reaction types of hydrazine-derived C-centered radicals (Figure 1c) are addition to C=C bonds followed by intermolecular trapping of second intermediate C-centered radical (alkene difunctionalization), addition to C=C bonds followed by intramolecular cyclization, and addition followed by oxidative dehydrogenation (formal hydrogen substitution reactions, most typical for heteroarenes). It should be noted that curved arrows in Figure 1c for cascade cyclizations do not imply a concerted process, but show sequential mechanistic steps (one step per each curved arrow).

## 2. Additions to the C=C Double Bonds

In the following chapter, we describe the addition of radicals, generated from the different hydrazine derivatives, to different substates with C=C and C≡C bonds and their heteroanalogs (e.g., hydrazones and isonitriles), followed by different types of radical trapping.

### 2.1. Additions to C=C Double Bonds Followed by Intermolecular Reactions of the Resultant Carbon-Centered Radicals

**Scheme 1:**
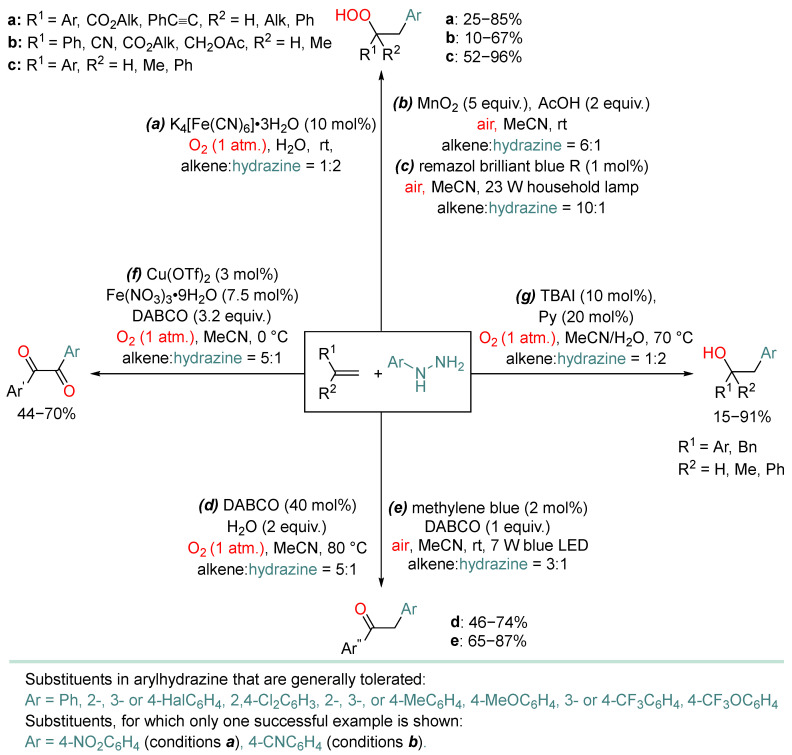
Alkene difunctionalization with arylhydrazines and oxygen.

Under aerobic conditions, the difunctionalization of alkenes with radicals typically yields oxygenated products such as ketones, alcohols or hydroperoxides. After the addition of a radical to C=C bond, the transient carbon-centered radical intermediate is intercepted by molecular oxygen. The reaction of alkenes with aryl radicals generated from arylhydrazines is no exception. The selectivity of the transformation is closely related to the structure of the substrate: 1,2-disubstituted alkenes form more stable peroxides, while alkenes with only one substituent at the double bond most often yield a mixture of hydroperoxide, ketone and a small amount of alcohol. The first examples of alkene hydroperoxidation with arylhydrazines used either K_4_[Fe(CN)_6_] as a catalyst (Scheme 1a) [45] or an excess of MnO_2_ oxidant (Scheme 1b) [46]. In both cases, formed hydroperoxides can be reduced to the corresponding alcohols by Na_2_S_2_O_3_. Reaction is applicable to electron-rich and electron-poor alkenes, including simple alkenes with unconjugated C=C bonds. Later, a transition-metal-free photochemical approach was developed using remazol brilliant blue R dye as photocatalyst (Scheme 1c) [47]; however, in this case, the scope of alkenes included mainly styrene derivatives, except for methyl acrylate and methyl methacrylate.

From styrenes, it is possible to obtain arylketones in good to excellent yields. The presence of a base is critical for the reaction’s success. In this case, DABCO (1,4-diazabicyclo[2.2.2]octane) serves this essential role in both chemical [48] and photochemical [49] approaches (Scheme 1d,e). In fact, without DABCO, arylketone is produced in 10% yield instead of 80% in optimal conditions [49]. Transition metal catalysis with Cu and Fe salts proved effective for the further oxidation of the second benzylic position of the resultant aryl ketone, yielding the 1,2-diketone (Scheme 1f) [48]. This transformation is also dependent on base: it deprotonates the intermediate arylketone, facilitating its further oxidation to diketone [48]. It should be noted that the base catalysis is quite typical for aerobic oxidation hydrazine derivatives, especially arylhydrazines, as will be shown later in the text.

The selective synthesis of the alcohol via hydroxyl-arylation of alkenes is slightly more complex, as it requires the reduction in hydroperoxides under overall oxidative conditions. Typically, this is accomplished by a two-step procedure that includes a hydroperoxide reduction step [45]. However, one elegant solution is the use of an I_2_/I^−^ redox system, with I^−^ and excessive arylhydrazine acting as reductants (Scheme 1g) [48]. In most cases for transformations **d**–**g**, vinyl arenes were used as one of the most effective aryl radical acceptors.

There are no special limitations on the nature of aryl substituent in the arylhydrazine for all mentioned in the Scheme 1 transformations. However, successful reactions using 4-NO_2_C_6_H_4_NHNH_2_ [45] and 4-CNC_6_H_4_NHNH_2_ [46] as substrates are each limited to a single example.

**Scheme 2:**
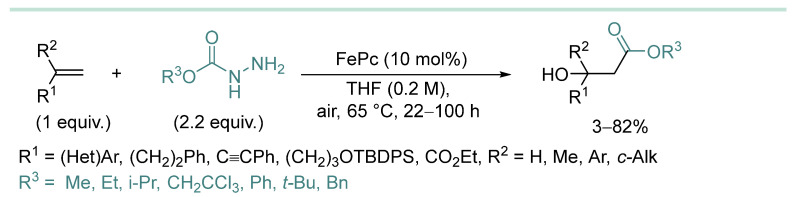
Alkene difunctionalization with carbazates.

The hydroxyl-acylation of 1,1-disubstituted alkenes with carbazates proceeds in the presence of the iron phthalocyanine (FePc) catalyst (Scheme 2) [50]. The formation of an alkoxycarbonyl radical from a carbazate under developed reaction conditions was confirmed by preparative trapping control experiment with TEMPO (yield 60%). The lowest yields were observed for R^3^ = Bn (3%) and *t*-Bu (7%) in carbazate, presumably, due to side process of decarboxylation favored by formation of stabilized Bn and *t*-Bu radicals. The reaction with other alkyl and phenyl carbazates generally proceeds in moderate to good yields (47–82%) [50]. FeCl_3_ also worked as a catalyst, although less effectively than FePc.

**Scheme 3:**
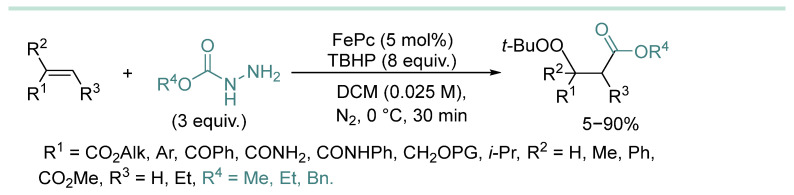
Alkene difunctionalization with carbazates and *tert*-butylhydroperoxide.

Under inert atmosphere, it is possible to introduce a variety of functional groups at the radical center formed upon addition of hydrazine-derived C-radical to the C=C bond. For example, the *tert*-butylperoxyl radical can intercept the intermediate carbon-centered radical (Scheme 3) [51]. In this reaction, *tert*-butyl peroxide serves a dual role, acting as both the oxidant and a coupling partner. Notably, most alkene substrates were vinylarenes or acrylate derivatives. As in the example presented above, the lowest yield (5%) was observed for the benzyl carbazate (R^4^ = Bn).

**Scheme 4:**
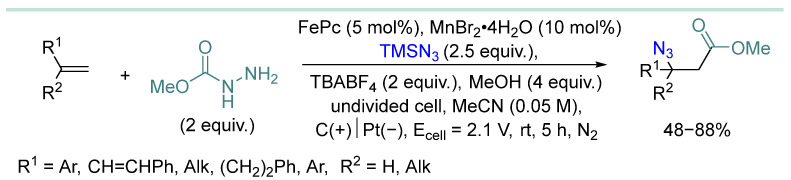
Alkene difunctionalization with carbazates and TMSN_3_.

The electrochemical difunctionalization of alkenes with the introduction of methoxycarbonyl and bioorthogonal azide groups was demonstrated employing methyl carbazate, TMSN_3_ and FePc/MnBr_2_ electrocatalytic system (Scheme 4) [52]. According to cyclic voltammetry and control experiments, FePc effectively catalyzes oxidative carbamoyl radical generation from carbazates (MnBr_2_ was ineffective). Presumably, azide group is transferred to the intermediate C-centered radical by M(III)-N_3_ species and MnBr_2_ enhances the efficiency of this step: slightly lower target product yield was observed in the absence of MnBr_2_ (74% instead of 87%). Notably, similar electrochemical Mn salt catalysis was used for alkene diazidation previously [53].

**Scheme 5:**
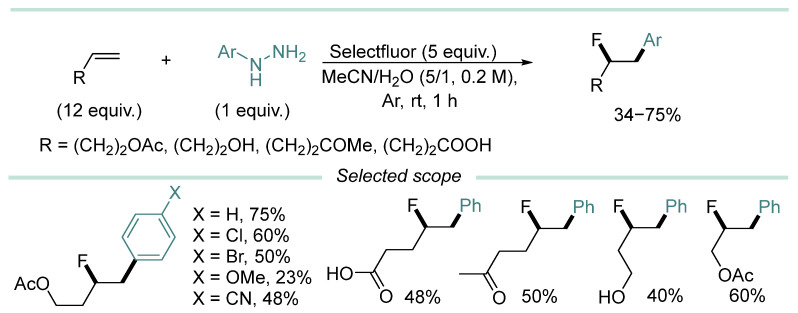
Alkene arylation-fluorination with arylhydrazines and Selectfluor.

Selectfluor was used as both oxidant for aryl radical generation from arylhydrazines and fluorinating agent (Scheme 5) [54,55]. However, the oxidative nature of Selectfluor has its downsides: it also participates in the formation of diazene by-products. It was later shown that the addition of three equivalents of anisole attenuates the reactivity of Selectfluor and improves the yields of the desired fluoride [55].

**Scheme 6:**
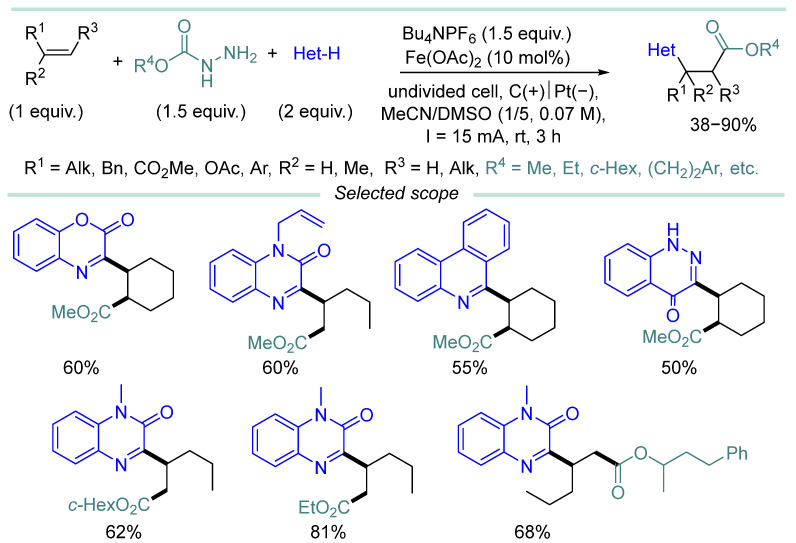
Alkene dicarbofunctionalization with carbazates and electron-deficient heteroarenes.

The combination of radical C–H functionalization of electron-deficient heteroarenes (the Minisci-type reaction) with alkene difunctionalization in a single step is a highly effective strategy for the rapid buildup of molecular complexity. Electrochemical approach enables facile alkene dicarbofunctionalization with carbazates and electron-deficient heteroarenes (Scheme 6) [56].

**Scheme 7:**
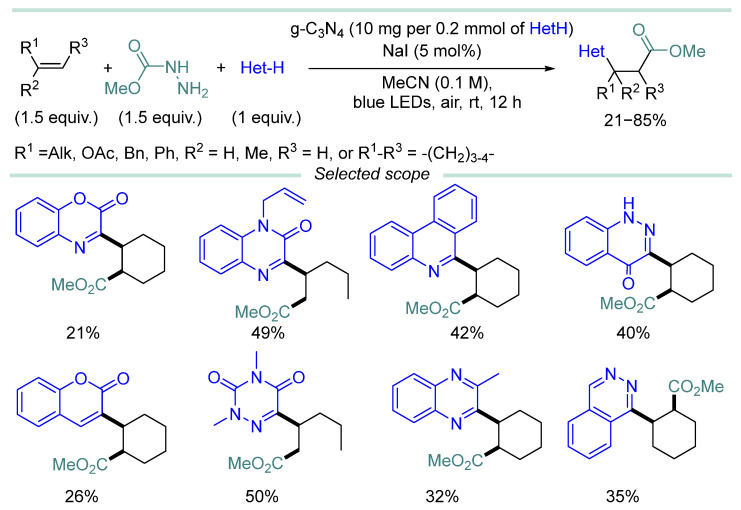
Difunctionalization of alkenes with heteroarenes and methyl carbazate in g-C_3_N_4_/NaI photocatalytic system.

Similar transformation was achieved employing the combination of heterogeneous photocatalyst g-C_3_N_4_ with NaI as I^−^/I• redox mediator oxygen from the air as the terminal oxidant [57]. Thus, this system enables the functionalization of different classes of heterocycles with carbon-centered radicals, created by the addition of carboxymethyl radicals generated from methyl carbazate to alkenes (Scheme 7). This approach is compatible with various functional groups, such as alkene or alkyne in the heterocycle. Although the main attention in the article is paid to the functionalization of quinoxalinones, a variety of chemically different heterocycles is tolerated under reaction conditions, including azauracil, cinnolin-4(1*H*)-one, isoquinoline, 2,3-diazonaphthalene, quinoxaline, phenanthridine, 2*H*-benzo[*b*][1,4]oxazin-2-one and coumarin. However, the yields for these substrates did not exceed 50%.

**Scheme 8:**
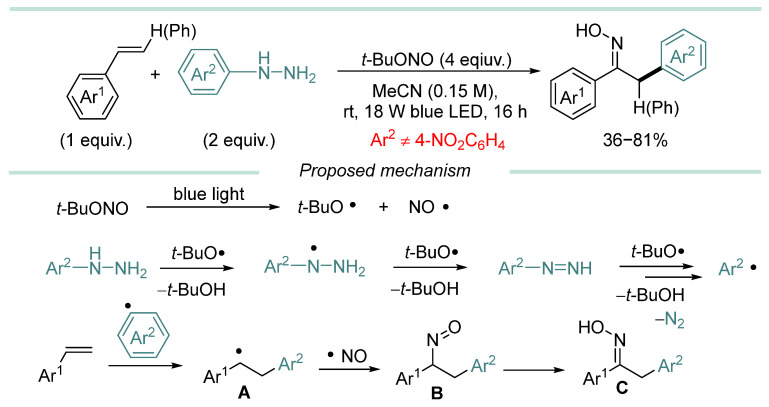
Difunctionalization of styrenes with arylhydrazines and *tert*-butyl nitrite. Red color represents substituents for which no target product was formed.

Vinylarene arylation-oximation was achieved employing *tert*-butyl nitrite photolysis by blue LED (Scheme 8) [58]. The photolysis of the *tert*-butyl nitrite gives two radicals—*tert*-butoxy and NO. Transient t-BuO• radical serves as the oxidant for arylhydrazine to form aryl radical (Ar^2^). Aryl radical Ar^2^ adds to vinylarene with the formation of benzylic radical **A**. NO plays the role of radical **A** scavenger, producing nitroso-substituted product **B**. In the case of styrene difunctionalization, the formed nitroso adduct **B** undergoes rapid isomerization to the corresponding oxime **C**. Among the limitations of this process is the inability to obtain the desired product with 4-nitrophenylhydrazine.

**Scheme 9:**
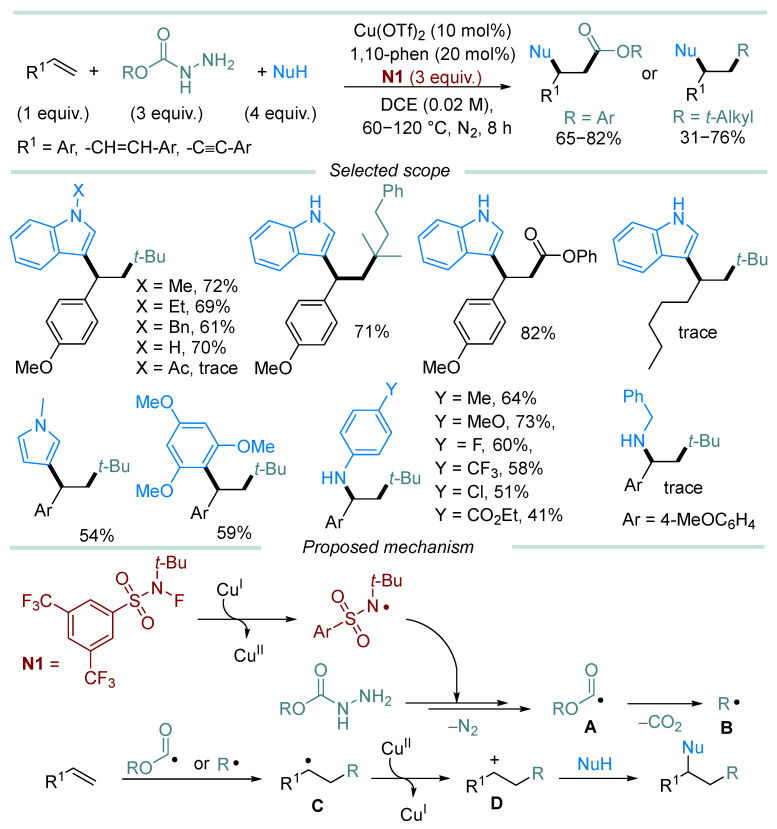
Difunctionalization of alkenes with carbazates as aryloxycarbonyl or *tert*-alkyl radical precursors and nucleophiles.

Another notable strategy to generate radicals from carbazates is the use of *N*-(*tert*-butyl)-*N*-fluoro-3,5-bis(trifluoromethyl)benzenesulfonamide (N1) as the HAT reagent (Scheme 9) [16]. Under moderate heating, carboxyalkyl radicals **A** generated in this way from *tert*-alkyl carbazates undergo decarboxylation to give *tert*-alkyl radicals **B**, whereas aryl carbazates give carboxyaryl radicals. The addition of thus formed radicals **A** or **B** to alkenes produces intermediate carbon-centered radicals **C**. The subsequent oxidation of carbon-centered radicals to carbocations **D** in the presence of Cu(II) makes possible coupling with indoles, *N*-methylpyrrole and 1,3,5-trimethoxybenzene as C-nucleophiles or with arylamines as *N*-nucleophiles.

Vinylazides are particularly versatile starting materials for radical transformations [59]. Upon addition of radical to the terminal position of the alkene they are transformed into transient carbon-centered radical species that are α-substituted with an azide functional group. This α-azido alkyl radical intermediate is unstable and rapidly undergoes extrusion of a dinitrogen molecule to form an iminyl radical. The resulting iminyl radical is a versatile intermediate that can undergo several transformations to a range of different products. Upon hydrogen atom abstraction it turns to imine, which can be hydrolyzed to ketone. The imine may tautomerize to form an enamine [60,61,62]. Radical–radical homocoupling of iminyl radical can produce an azine.

**Scheme 10:**
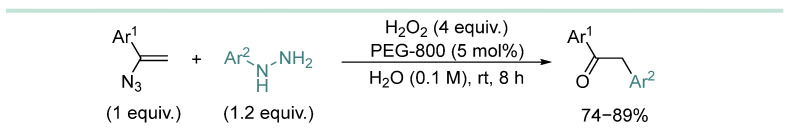
Radical arylation of vinylazides by arylhydrazines in aqueous media.

In the developed method [63] aryl radical generated from arylhydrazine in the aqueous media employing H_2_O_2_ as oxidant adds to the vinyl azide, initiating the cascade described above (Scheme 10). Under aqueous conditions, the final product is the ketone, resulting from the hydrolysis of the intermediate imine. A further significant finding of this study is a novel method for generating aryl radicals using hydrogen peroxide—an abundant and environmentally benign reagent—in combination with PEG-800. Taking into account its scalability, cost efficiency, the use of water as a solvent, and hydrogen peroxide as an oxidant (which produces only water as a by-product), the developed procedure represents not only a sustainable method for synthesizing arylketones but also a promising strategy for aryl radical addition to different substrates.

**Scheme 11:**
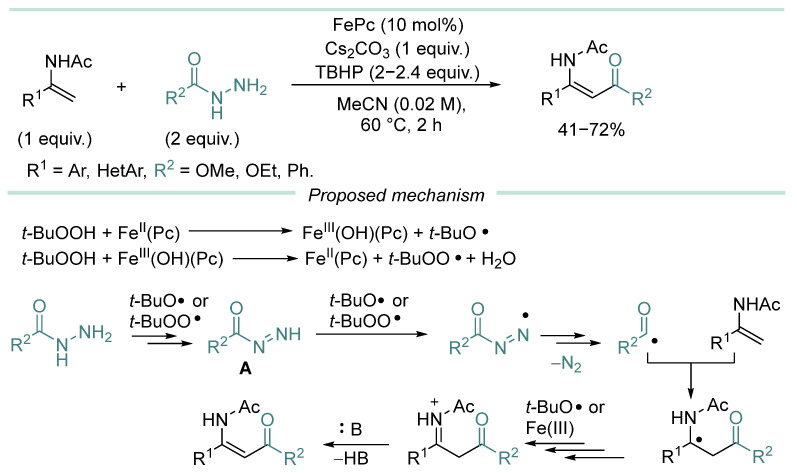
Iron phthalocyanine (FePc) catalyzed alkoxy-carbonylation of *N*-vinylacetamides with carbazates.

*N*-vinylacetamides are efficient radical acceptors, which make possible realization of formal hydrogen substitution by radical (Scheme 11) instead of C=C bond difunctionalization reactions discussed above (Scheme 2, Scheme 3, Scheme 4, Scheme 5, Scheme 6, Scheme 7, Scheme 8, Scheme 9 and Scheme 10). Acyl radicals for this process were generated from acylhydrazides and carbazates using *tert*-butyl hydroperoxide (TBHP) as an oxidant in the presence of iron phthalocyanine (FePc) as the catalyst [64]. Iron phthalocyanine (FePc) catalyzes the formation of reactive *tert*-butyl peroxyl or *tert*-butoxyl radicals from TBHP, which in turn drive oxidation of carbazates or acyl hydrazides to the corresponding carbon-centered radicals **A**. These radicals **A** add to enamines to form alkoxycarbonylated products (Scheme 10). In contrast to previously discussed processes involving FePc-catalyzed oxidation of hydrazine derivatives (Scheme 2, Scheme 3 and Scheme 4), the base (Cs_2_CO_3_) was used in the present case. In the absence of the base, yield of a model target product was lower (41% instead of 68%). The supposed role of the base is the deprotonation of the intermediate iminyl cation **B**.

### 2.2. Additions to Acrylamides Followed by Intramolecular Cyclizations

*N*-acrylamides are privileged substrates in radical-mediated cascade additions–cyclizations, giving access to structurally diverse *N*-heterocycles. Two main types of such processes are discussed in this section: cyclization to form oxindoles and cyclization to form isoquinolinone core.

#### 2.2.1. Type 1—Cyclization to Oxindoles

The synthesis of oxindoles via radical-mediated cyclization of *N*-acrylamides now can be considered as well-explored model transformation for assessing the performance of various radical initiators and oxidative systems. In Table 1, various oxidative systems are summarized. Entries 1–5 employ a peroxide-based terminal oxidant—either an organic peroxide or an inorganic persulfate—used in significant excess (3–6 equivalents). Persulfates are cost-effective and strong oxidants which find broad usage in modern organic synthesis involving oxidative generation of free radicals [65], including many examples from the present review. It is interesting to note that in oxidation of RNHNH_2_, both strongly oxidative S_2_O_8_^2−^ and much milder oxidant TBHP are used in many cases for the oxidation of the same hydrazine classes. Entries 6–7 represent electrochemical methods, wherein anodic oxidation generates radicals from arylhydrazines or carbazates. A common requirement across all methods is the use of an excess of the hydrazine derivative (2–4 equivalents), with arylhydrazines requiring a comparatively lower stoichiometry in some cases (1.5 equivalents, entry 6). This trend, reflecting the dependence of reaction efficiency on the hydrazine derivative’s structure, will persist throughout this review.

#### 2.2.2. Type 2—Cyclization to Isoqunolinones

Indolo[2,1-α]isoquinolines represent an important class of *N*-heterocycles, which widely exist in natural products, pharmaceuticals and material science [73]. As oxindoles, they can also be obtained by cyclization of *N*-acrylamides using methods very similar to those described above in Table 1. TBHP or persulfate is typically used as the terminal oxidant in a large excess (3–8 equivalents, Table 2). Carbazates, acyl hydrazides and *tert*-butylhydrazine are tolerated in this transformation.

**Scheme 12:**
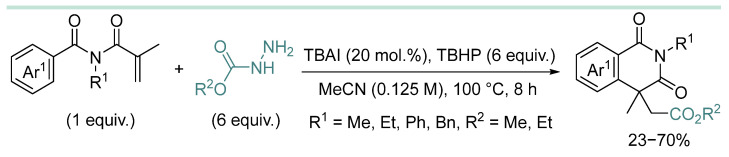
The synthesis of isoquinolinedione derivatives by alkoxycarbonylation of *N*-acrylamides.

*N*-benzoyl-*N*-acrylamides readily undergo intramolecular cyclization to form isoquinolinediones, employing the TBAI/TBHP oxidative system (Scheme 12) [78].

**Scheme 13:**
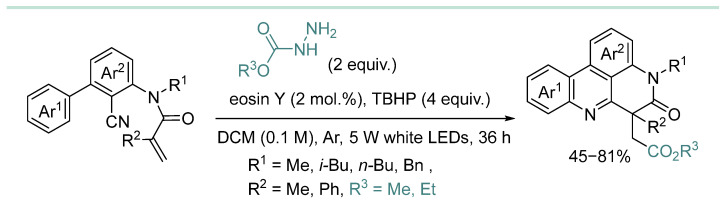
The synthesis of phenanthridine derivatives by alkoxycarbonylation of nitrile-containing *N*-acrylamides.

The more elaborate heterocyclic cores can be achieved by this technic using more complex substrates, where the nitrile group is appended to a benzene ring (Scheme 13) [79]. In these systems, the carbon-centered radical generated in situ does not add to the aromatic ring but instead adds to the nitrile group. This addition produces an iminyl radical intermediate, which subsequently undergoes intramolecular cyclization to form a quinoline system.

**Scheme 14:**
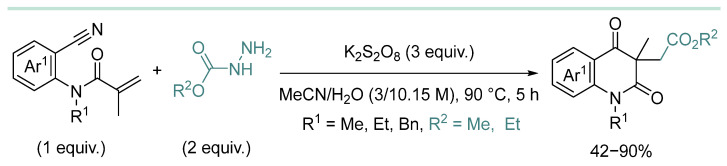
The synthesis of isoquinolinedione by alkoxycarbonylation of *N*-acrylamides.

Similarly, various quinoline-2,4-diones can be achieved by the cyclizaition of *N*-(2-cyanoaryl)-*N*-methacrylamides using potassium persulfate as oxidant (Scheme 14) [80]. This strategy is readily scalable and allows for facile derivatization (e.g., selective hydrolysis, reduction, dealkylation), making it highly applicable for the synthesis of diverse valuable molecules.

**Scheme 15:**
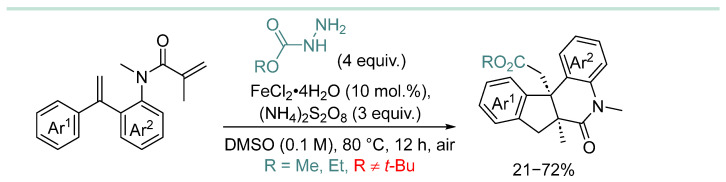
Iron-catalyzed radical cascade cyclization of dienes initiated by an alkoxycarbonyl radical. Red color represents substituents for which no target product was formed.

The next example (Scheme 15) [81] differs from the additions to *N*-acrylamides discussed above in one principal step: here, carbazate-derived carbon-centered radical first attack the 1,1-diaryethylene double bond, and then the so-formed carbon-centered radical intramolecularly adds to the *N*-acrylamide moiety. This reactivity demonstrates a pronounced chemoselectivity, where the alkoxycarbonyl radical preferentially attacks the neutral vinyl moiety (1,1-diarylethylene) over the electron-deficient alkene (*N*-acrylamide). Authors note that according to computational data from the literature, alkoxycarbonyl radicals are less nucleophilic compared to acyl radicals and can generally react as ambiphilic or electrophilic radicals, which make their reactivity in the discussed case not obvious [81]. However, the reaction was also found to be highly sensitive to steric hindrance. This is evidenced by the complete absence of product formation for substrates featuring *ortho*-substituted aryl rings.

## 3. Addition to Hydrazones

**Scheme 16:**
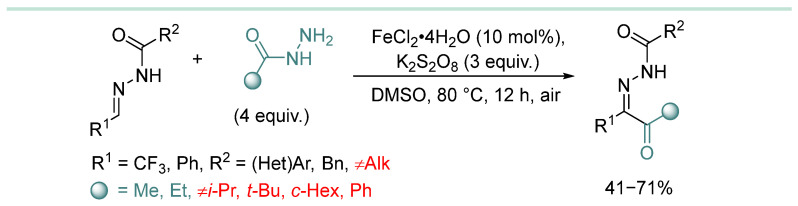
C-acylation of hydrazones with acyl hydrazides. Red color represents substituents for which no target product was formed.

The Fe(II)/K_2_S_2_O_8_ oxidative system enables the C-acylation of *N*-acylhydrazones using acyl hydrazides (Scheme 16) [82]. This finding expands the scope of radical acylations beyond traditional alkene substrates to include imine systems. However, the scope is limited to acetyl and propionyl hydrazides.

**Scheme 17:**
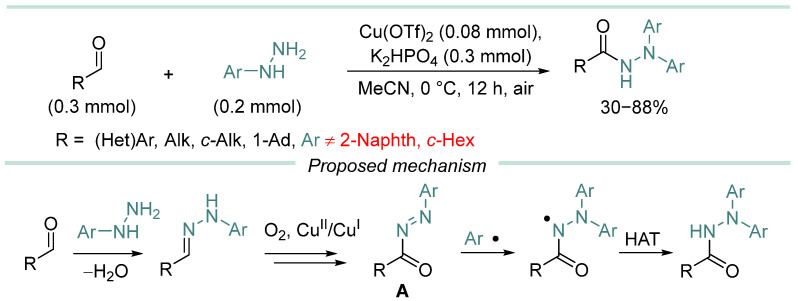
Synthesis of *N*,*N*-diarylhydrazides from aldehydes and arylhydrazines via the formation of hydrazones in situ. Red color represents substituents for which no target product was formed.

The hydrazones generated from arylhydrazines and carbonyl compounds are prone to oxidation under the conditions used for the generation of aryl radicals from starting arylhydrazines. This process reaction leads to an unexpected product containing two aryl fragments (Scheme 17) [83]. Specifically, the condensation between aldehyde and arylhydrazine produces intermediate hydrazone, which is oxidized in the presence of oxygen and a Cu(II) salt to diazene A. This diazene A can then readily intercept the aryl radicals generated by the same oxidative system. The formation of intermediate diazene **A** was not detected under the reaction conditions. However, when **A** was synthesized separately and introduced into the reaction with arylhydrazine in the presence of Cu(OTf)_2_/K_2_HPO_4_/air system, the *N*,*N*-diarylhydrazide was formed successfully.

## 4. Addition to Alkynes

**Scheme 18:**
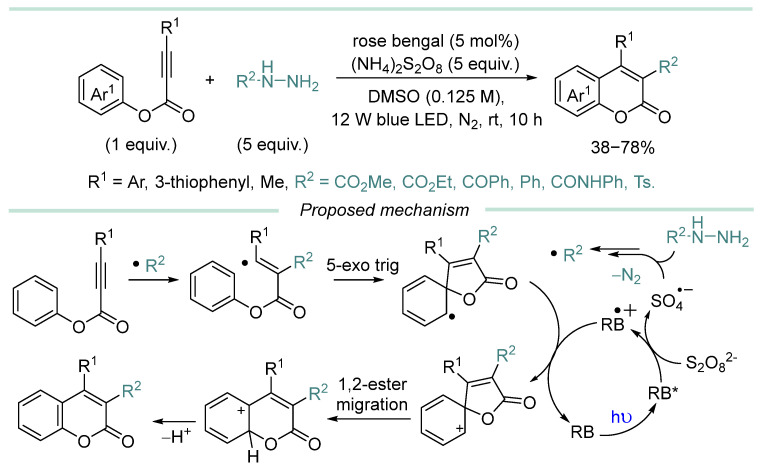
The synthesis of coumarin derivatives from aryl alkynoates and hydrazines.

Reactions of hydrazine-derived radicals with alkynes are much less explored compared to similar alkene functionalization processes, which can be attributed to lower reactivity of alkynes compared to alkenes. The notable example is the effective construction of coumarins from aryl alkynoates and hydrazines was demonstrated employing rose bengal visible light photocatalysis and (NH_4_)_2_S_2_O_8_ as oxidant (Scheme 18) [84]. This method tolerates a wide variety of hydrazine derivatives: carbazates, acyl hydrazides, arylhydrazines, hydrazine carboxamides and sulfonyl hydrazides. It gives the opportunity to synthesize coumarins with a variety of substitution patterns.

**Scheme 19:**
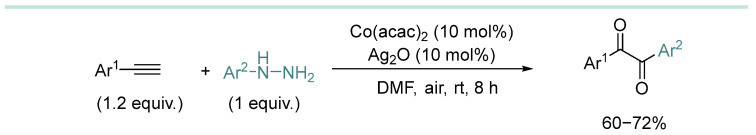
Co-catalyzed functionalization of alkynes with phenylhydrazines.

The Co(acac)_2_/Ag_2_O-catalyzed reaction between alkynes and arylhydrazines under aerobic conditions yielded vicinal diketones (Scheme 19) [85]. Strong electron-withdrawing substituents on either the arylhydrazine (*p*-CN, *m*-NO_2_) or the acetylene (pyridine-2-yl) were not tolerated in this method.

## 5. Addition to Isonitriles

The role of isonitriles as radical acceptors is widely recognized in modern synthetic organic chemistry, especially in the synthesis of nitrogen heterocycles by cascade radical addition reactions [86]. The addition of a radical to the isonitrile leads to an imidolyl radical, which can undergo a wide range of intramolecular cyclization reactions, leading to numerous pharmaceutically relevant *N*-heterocycles such as phenanthridines, quinolines, isoquinolines, quinoxalines and indoles. For example, one of the most frequent reactions in the literature is the reaction of 2-isocyanobiphenyls to form phenanthridines (Table 3). Both electron-donating (Me, MeO) and electron-withdrawing groups (F, Cl, Ac, CO_2_Me, CN, CF_3_, etc.) are well tolerated in such transformations. As a rule, substrates bearing *ortho* substituents show slightly lower reactivity due to steric effects. 2-Isocyanobiphenyls bearing *meta*-substituents yield mixtures of two regioisomers with a typical ratio ranging from 2:1 to 2.7:1.

As with other reactions involving hydrazine derivatives, oxidative systems with transition metal salt/TBHP or TBAI/TBHP are the most common for radical generation from carbazates (entries 1–3), while photocatalytic systems with excess base were successfully employed for the radical generation from aryl and alkylhydrazines (entry 4).

**Scheme 20:**
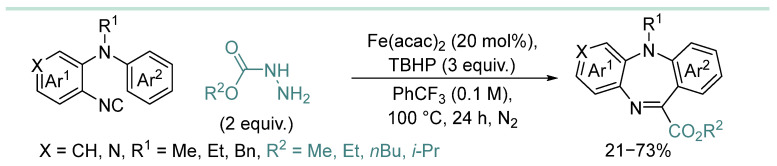
The synthesis of benzodiazepine heterocycles from *o*-isocyanodiaryl amines.

The radical addition to isonitriles is not limited to forming six-membered rings. For instance, reaction with *o*-isocyanodiaryl amines [92] provides access to seven-membered dibenzodiazepine heterocycles (Scheme 20), a scaffold with potential biological and pharmacological activities. The conditions are similar to the conditions of runs 1 and 2 in Table 3; however, the PhCF_3_ solvent here was slightly more efficient than PhF (see SI for the paper [92]). These results can be attributed to the higher reaction temperature (100 °C instead of 80 °C) due to higher boiling point of PhCF_3_. Reaction proceeded with phenylhydrazine instead of carbazates, though poor yield was observed (20%).

**Scheme 21:**
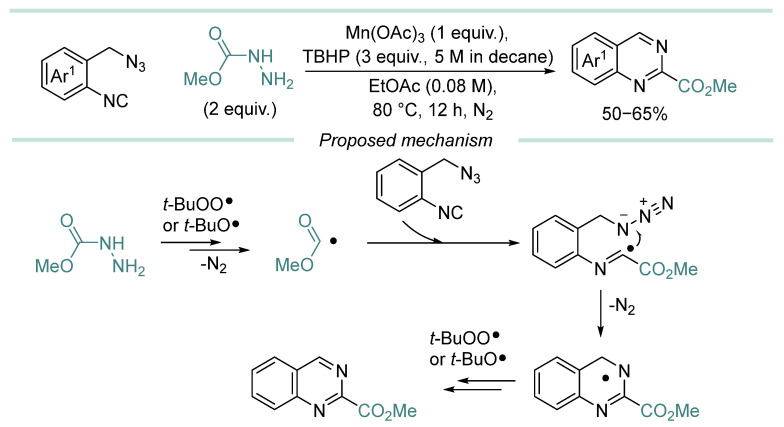
Radical cyclization of 2-(azidomethyl)phenyl isocyanides to methyl quinazoline-2-carboxylates employing methyl carbazate as methoxycarbonyl radical precursor.

The presence of an azide group in the arylisocyanide enables a novel synthetic route. 2-(Azidomethyl)phenyl isocyanides undergo intramolecular cyclization to form methyl quinazoline-2-carboxylates in good yields (Scheme 21) [93]. Substrate electronic effects modulate the reaction efficiency, with electron-donating groups on the benzene ring affording slightly superior yields (~60%) compared to electron-withdrawing groups (~50%). It should be noted that in addition to the proposed mechanism involving the radical attack on the nitrogen atom of the azide group (Scheme 21), alternative pathways can be expected involving the hydrogen atom abstraction from the α-position relative to the azide group, leading to N_2_ extrusion and iminyl radical formation [94,95,96].

**Scheme 22:**
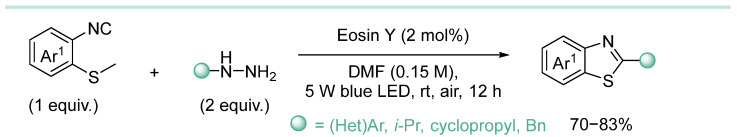
The synthesis of 2-aryl or 2-alkyl benzothiazoles from (2-isocyanophenyl)(methyl)sulfanes and hydrazines.

Isocyanides provide a versatile platform for synthesizing heterocycles, including 5-, 6- and 7-membered rings. Among these applications, the preparation of 2-aryl and 2-alkyl benzothiazoles is highly attractive because it proceeds with ease under mild, metal-free photochemical conditions (Scheme 22) [97]. Notably, this reaction is applicable to alkylhydrazines and benzylhydrazine, in addition to (hetero)arylhydrazines used in most works discussed above.

**Scheme 23:**
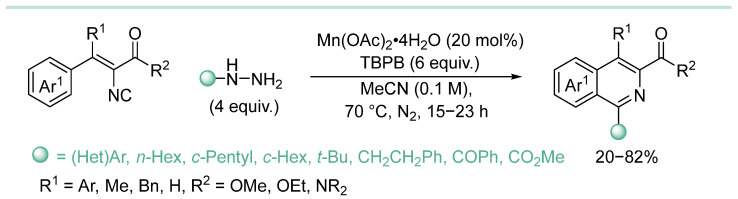
The synthesis of isoquinolines from vinyl isocyanides and hydrazines.

Vinyl isocyanides serve as effective precursors for the synthesis of isoquinolines via radical addition [98]. This reaction demonstrates a broad substrate scope for hydrazine derivatives, showing high compatibility with both arylhydrazines and alkylhydrazines, while acylhydrazides and carbazates give moderate yields. Notably, the R^1^ substituent is not limited to aryl groups but can also be a hydrogen atom, significantly enhancing the versatility of this transformation (Scheme 23).

## 6. Additions to (Hetero)Arenes with Formal Hydrogen Substitution

Historically, the first examples of arene functionalization with hydrazine-derived carbon-centered radicals are related to the functionalization of the benzene [99], furan or thiophene [100]. These transformations required large excesses of the substrate—at least dozens of mL of arene to 1 mmol of arylhydrazine [101]. Although this procedure may seem impractical, they laid the foundation for the development of other coupling reactions with less demanding substrates. As we will see, the oxidants used in these pioneering articles, Mn(OAc)_3_ and KMnO_4_, are widely implemented for the radical arylation of different arenes.

The *N*-heterocycles occupy an important place in medicinal chemistry. They are found in essential biomolecules, such as the nucleotide bases, some amino acids, plant growth regulators, biocidal substances produced by various organisms, etc. It is therefore not surprising that interest in the functionalization of *N*-heterocycles to create new promising biologically active compounds is growing. Among the radical approaches to heterocycle functionalization, the most widely used is the Minisci-type process: the addition of carbon-centered radicals to heterocycles followed by oxidative re-aromatization. The original Minisci reaction uses carboxylic acids as radical precursors via oxidative decarboxylation, but decades of research have yielded other precursors, including hydrazine derivatives.

Given the vast variety of *N*-heterocycle classes, this review will first deal with six-membered and condensed heterocycles with 1–3 nitrogen atoms, before moving on to special classes that are known to be more easily functionalized.

### 6.1. Pyridine-Type N-Heterocycles: Pyridines, Quinolines, Quinoxaline, Isoquinoline, etc.

**Scheme 24:**
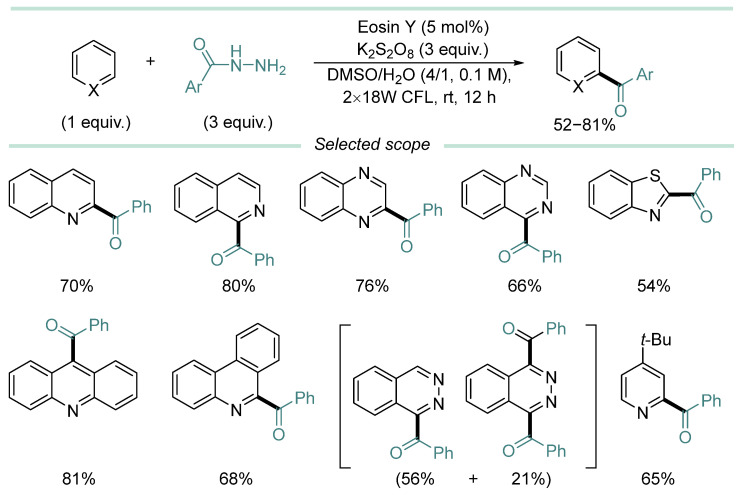
Photochemical C–H aroylation of different classes of *N*-heterocycles by aroylhydrazines.

A photochemical approach employing K_2_S_2_O_8_ as the terminal oxidant enables the aroylation of a wide variety of *N*-heterocycles by aromatic acyl hydrazides [102]. The *N*-heterocycles include isoquinoline, quinoline, phenanthridine, dihydroacridine, quinoxaline and phthalazine (Scheme 24). The reaction can be successfully scaled up to 5 mmol of substrate.

**Scheme 25:**
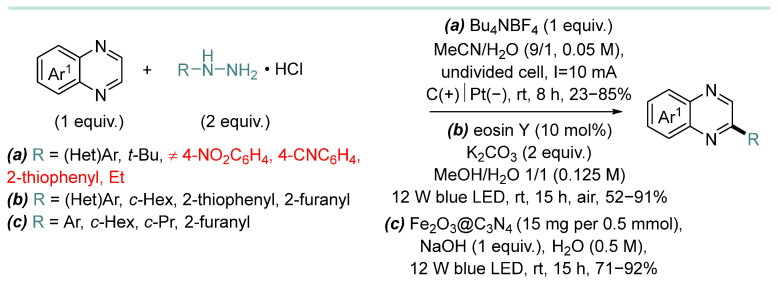
C–H arylation of quinoxalines with arylhydrazine hydrochlorides. Red color represents substituents for which no target product was formed.

An electrochemical method for the functionalization of quinoxaline and its derivatives was developed, proceeding without any redox catalyst [103]. Direct electrolysis in a MeCN/H_2_O solvent system within an undivided cell produced aryl- and *tert*-butyl-functionalized heterocycles in moderate to good yields (Scheme 25, conditions a). This technique is also applicable for the functionalization of quinoxaline-2(1*H*)-ones (see Section 6.3). A complementary approach, suited for hydrazines beyond the scope of the previous method such as 2-thiophenylhydrazine, uses photochemical conditions with eosin Y as the photocatalyst and K_2_CO_3_ as a base (Scheme 25, conditions b) [104]. Most recently, the heterogeneous photocatalytic system Fe_2_O_3_@C_3_N_4_ was demonstrated as an efficient method for the same transformation. (Scheme 25, conditions c) [105].

**Scheme 26:**
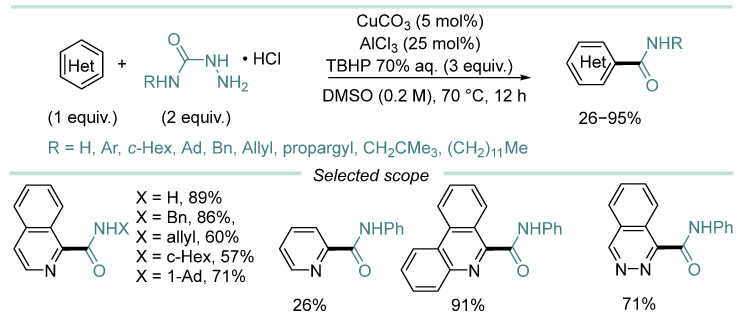
Functionalization of heteroarenes with hydrazine carboxamides.

The reaction of *N*-heterocycles with hydrazine carboxamides produced the corresponding heteroaromatic amides (Scheme 26) [106]. This method represents a facile, single-step synthesis for heteroaromatic amides bearing zero or one substituent on the nitrogen atom. In contrast, *N*,*N*-disubstituted hydrazine carboxamide hydrochlorides did not produce target amides, probably due to the steric repulsion imposed by the *N*-substituents. AlCl_3_ acts as a Lewis acid. It coordinates with the heterocyclic substrate, thereby enhancing its electrophilicity and facilitating subsequent attack by nucleophilic radical.

### 6.2. Imidazo[1,2-a]pyridines

Imidazo[1,2-*a*]pyridines are a class of *N*-heterocycles with wide applications in organic synthesis and pharmaceutical chemistry [107]. The radical functionalization of these heterocycles is one of the most efficient strategies for obtaining diverse imidazo[1,2-*a*]pyridine derivatives. Among these reactions, functionalization at the C3 position is the most commonly reported. Table 4 summarized such reactions with hydrazine-derived radicals.

**Scheme 27:**
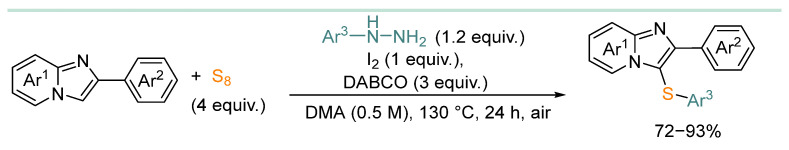
The synthesis of 3-sulfenylated imidazo[1,2-*a*]pyridines using elemental sulfur and arylhydrazine hydrochloride.

In addition to direct coupling, radicals from hydrazides can also undergo a recently discovered reaction where elemental sulfur incorporates into the C-3 position of the imidazo[1,2-*a*]pyridine (Scheme 27) [112].

**Scheme 28:**
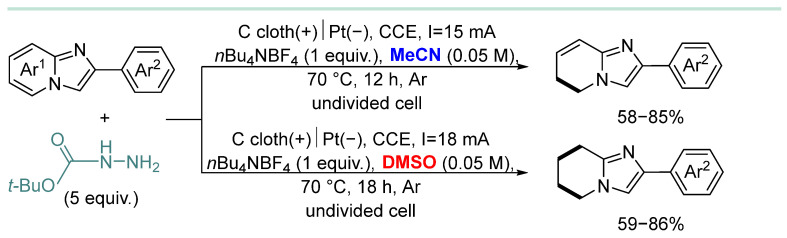
Electrochemical hydrogenation of imidazopyridines with *tert*-butyl carbazate. Solvents MeCN and DMSO are highlighted as the solvent nature determines the main product.

It turns out that *tert*-butyl carbazate can be an unusual reducing agent [113]. It was shown that under electrochemical conditions it can reduce imidazopyridines either to dihydroimidazo[1,2-*a*]pyridine (in MeCN) or to tetrahydroimidazo[1,2-*a*]pyridine (in DMSO) (Scheme 28).

### 6.3. Quinoxalin-2(1H)-Ones

In recent years, radical reactions using quinoxalin-2(1*H*)-ones as substrates have gained increasing interest [114]. The ease of radical functionalization at the C3 position is driven by rapid and irreversible re-aromatization of the initial radical adduct to a thermodynamically stable quinoxalin-2(1*H*)-one (Table 5). In addition to traditional oxidative systems based on persulfates or organic peroxides (conditions 1–3), the reaction effectively proceeds under photochemical conditions with a homogeneous organic photocatalyst (conditions 4–5), or with a heterogeneous one (conditions 6–8), or even without a photocatalyst under purple light irradiation (conditions 11–12). Notably, the latter conditions are one of the rare examples where the substrate and hydrazine derivative are taken in a stoichiometric ratio. Electrochemical conditions are also suitable for this transformation (conditions 13–14). The reaction tolerates *N*-allyl and *N*-propargyl groups and is successful even for the unsubstituted quinoxalin-2(1*H*)-one.

Although most methods for generating radicals from arylhydrazines require a base (e.g., DBU, [122] K_2_CO_3_ [120]), some proceed under base-free conditions. In one case, a hydrochloric acid was used instead [123]. It was shown that irradiation of HCl with blue light under aerobic conditions produced highly reactive chlorine radicals that oxidized the hydrazine. In another example [124], it was proposed that under purple LED irradiation, quinoxalinones act as photosensitizers. They mediate an energy transfer process with ground-state triplet oxygen (^3^O_2_) to form singlet oxygen (^1^O_2_), which, along with superoxide (O_2_^−^) and peroxide (HO_2_·) radicals, oxidizes a hydrazine.

**Scheme 29:**
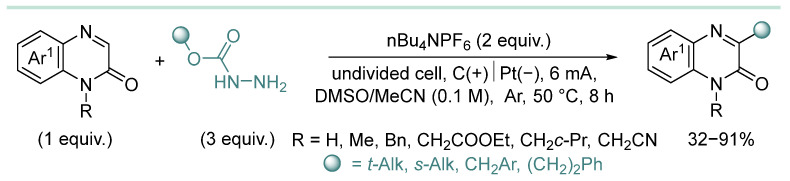
Electrochemical alkylation of quinoxalinones with carbazates.

The electrochemical decarboxylative oxidation of carbazates opens a new pathway for radical alkylation of various substrates, including quinoxalinones (Scheme 29) [127]. The range of suitable carbazates includes secondary and tertiary alkyl derivatives, while the use of methyl carbazate enables methoxycarbonylation [17,127]. These conditions are also applicable to the other electron-deficient heteroarenes, such as benzoquinoxalinone, pyrazinone, quinazolinone, isoquinoline, phthalazine, quinazoline and phenanthridine. Benzofuran and benzothiophene were not compatible with this method.

**Scheme 30:**
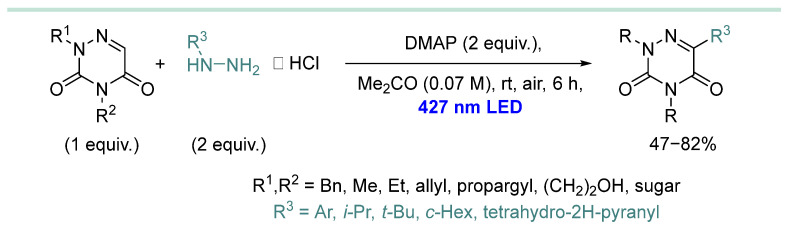
Photochemical functionalization of azauracils with hydrazines.

Structurally related class of heterocycles—azauracils—can also be functionalized with hydrazine derivatives (Scheme 30). Both arylation and alkylation of azauracils proceed under visible light in the presence of dimethylaminopyridine base (DMAP) [128].

### 6.4. 2H-Indazoles

2*H*-Indazoles are an important class of nitrogen-containing heterocycles, frequently found in bioactive natural products and drugs [129]. Late-stage C–H functionalization is a powerful strategy for rapidly increasing their structural complexity. The radical C3-functionalization of 2*H*-indazoles (Table 6) follows principles similar to the heterocycles discussed above, with most of the methods being identical.

The photochemical reaction of 2*H*-indazole with arylhydrazine [132] eloquently demonstrates the robustness of the process. Reaction proceeds in moderate to good yields with various photocatalysts (4CzIPN, rose Bengal, eosin Y, rhodamine, etc.) or even without one (33% yield with light and 17% in the dark). The reaction is also compatible with a range of bases (NaOH, *t*-BuOK, DIPEA, NEt_3_, DBU, pyridine).

### 6.5. Quinoline-N-Oxides

**Scheme 31:**
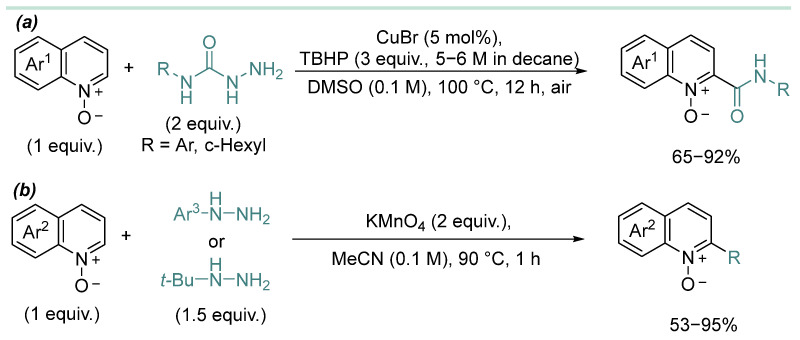
***(a)*** The C–H functionalization of quinoline-*N*-oxides with hydrazine carboxamides. ***(b)*** The C–H functionalization of quinoline-*N*-oxides with arylhydrazines or *tert*-butylhydrazine.

The reaction of quinoline-*N*-oxides with carbon-centered radicals proceeds with retention of the *N*-oxide group. Both hydrazine carboxamides [133] and arylhydrazines [134] can serve as radical sources. In the case of hydrazine carboxamides, the oxidative system is typical for reactions with other heterocycles—TBHP with transition metal catalyst CuBr. Notably, no reaction occurred with some 6-substituted quinoline-*N*-oxides (6-methoxyquinoline *N*-oxide, 6-methylquinoline *N*-oxide and 6-(methoxycarbonyl)quinoline *N*-oxide), but this phenomenon was not discussed in detail. Also, no product was obtained with 3-bromoquinoline *N*-oxide, probably due to the steric repulsion (Scheme 31a). KMnO_4_ in MeCN was used for arylation and *tert*-butylation of quinoline *N*-oxides by corresponding hydrazines (Scheme 31b) [134]. However, it should be noted that *tert*-butylhydrazine was used for the synthesis of only one example: 2-*tert*-butylquinoline *N*-oxide was isolated in 82% yield. Highly electron-deficient 6-nitroquinoline-*N*-oxide cannot be arylated in this manner [134].

### 6.6. Intermolecular Functionalization of Biaryls

**Scheme 32:**
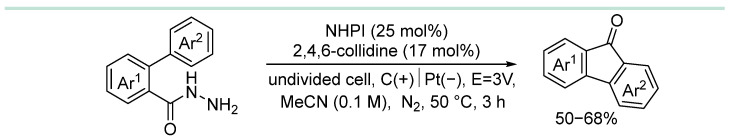
The synthesis of fluorenones by the intramolecular cyclization of o-aryl benzoylhydrazides.

While unactivated benzene rings are generally poor radical acceptors, they can effectively undergo intermolecular addition by acyl radicals when the outcome of the reaction is the formation of a stable five- or six-membered ring. A representative example is the cyclization of *ortho*-aryl benzoylhydrazides, which proceeds efficiently to yield fluorenone derivatives under constant voltage electrolysis conditions (Scheme 32) [135]. *N*-Hydroxyphthalimide (NHPI) serves as redox organocatalyst, and bases such as 2,4,6-collidine are known to facilitate anodic oxidation of NHPI to phthalimide-*N*-oxyl radicals. At the anode, it is oxidized to the phthalimide-*N*-oxyl radical, a potent hydrogen atom abstractor. This radical oxidizes the hydrazine in several steps, leading to an acyl radical, which subsequently undergoes intermolecular cyclization. The reaction proceeds in good yields with electron-donating substituents on the attacked benzene ring, while electron-withdrawing groups (CN, NO_2_) lead to the decrease in yields.

### 6.7. Functionalization of 1,4-Benzoquinones and 1,4-Naphthoquinones

Naphthoquinones are biologically significant compounds, valued in synthetic and medicinal chemistry. They exhibit a wide range of activities, including antibiotic, antifungal, anti-inflammatory, anti-allergic, apoptotic and antithrombotic effects [136]. The generation of aryl radicals from arylhydrazines offers an attractive strategy for the direct arylation of 1,4-benzoquinones and 1,4-naphthoquinones. This approach has been the focus of several recent methodological developments (Table 7). Both traditional strategies based on aerobic oxidation under basic conditions, as well as more elaborate systems employing electric current, iodine, or hypervalent iodine compounds, have proven effective for the functionalization of quinones bearing a wide range of substituents.

### 6.8. Functionalization of Anilines

The functionalization of aminoarenes stands out from other arene functionalization reactions in one principal aspect: for a successful reaction, up to 20 equivalents excess of the aminoarene are needed. Pioneering researchers employed large excesses of transition metal oxidant MnO_2_ (Table 8, entry 1) [141]. Furan, phenol and anisole also undergo arylation under this conditions [141]. Greener catalytic approaches have been developed later (entries 2–7). Arylhydrazine hydrochlorides were used as more stable and convenient reagents instead of free aryl hydrazines, employing 1 M aq. NaOH as a base (Table 8, entry 2) [142]. It should be noted that reaction proceeds under basic conditions, as a arylhydrazine hydrochloride was added to the mixture of an aniline derivative and 1 M NaOH gradually (10 batches over 9 h) in order to suppress participation of aryl radicals in hydrogen atom abstraction from the hydrazine instead of addition to aniline π-system [142]. Aminopyridines, aminoquinolines and aminopyrazines are tolerated under simple aerobic conditions with an excess of K_2_CO_3_ as a base and no additional catalyst (Table 8, conditions 3–7) [143,144]. Regioselectivity of arylation depends on substituents and generally proceeds with participation of unsubstituted *ortho*- or *para*- position to aminogroup; however, acidic conditions can lead to the formation of meta-substituted side product [141]. Unsubstituted aniline was phenylated at *ortho*-position employing CoPc/air system (62% yield) [145] or NaOH/air system (45% yield) [146].

### 6.9. Functionalization of Other Heterocycles

**Scheme 33:**
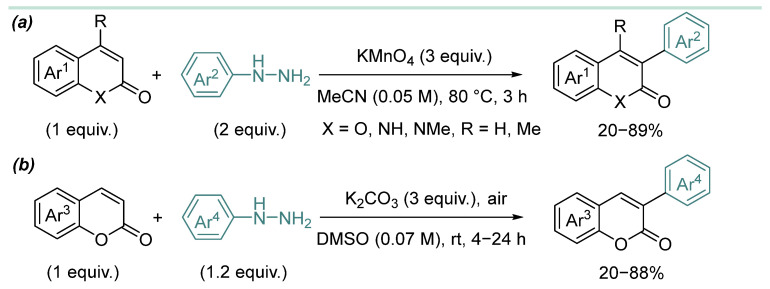
Radical arylation of coumarins with arylhydrazines: ***(a)*** with the KMnO_4_ as oxidant, ***(b)*** under aerobic conditions.

Arylation of coumarins and quinolinones with arylhydrazines was achieved using potassium permanganate as an oxidant (Scheme 33a) [147]. The method is applicable to free hydrazines, whereas for hydrazine hydrochloride salts, pretreatment with NaOH is required. Both arylhydrazines with electron-donating and electron-withdrawing substituents are tolerated in the reaction. However, bulky *ortho*-substituents, such as bromine, prevented the formation of the desired product, presumably due to steric hindrance. It is worth mentioning that *tert*-butylhydrazine was also used in the reaction, and the expected product was obtained in 35% yield. Another approach to this transformation uses a base under aerobic conditions (Scheme 33b) [148]. In this case, the reaction is more sensitive to substituents in the coumarin core; for example, no product was observed with 6-nitrocoumarin. Furthermore, no C-4 functionalization occurred with C-3 arylated coumarins. The procedure is also applicable to 2-pyridones, with or without N–H protection.

**Scheme 34:**
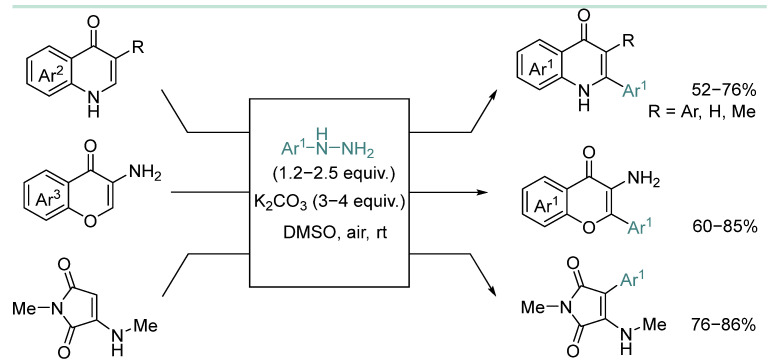
Radical arylation of quinoline-4-ones, 3-aminochromones 2-aminomaleimides with arylhydrazines.

The radical addition of aryl radicals, generated in situ from arylhydrazines, to a heterocycles, containing a cyclic enamine fragment, e.g., quinolin-4-ones [149], demonstrates that the presence of a base is sufficient for the radical generation from these hydrazine precursors. A strong terminal oxidant, such as a peroxide, is not always required (Scheme 34). Nearly identical conditions were later implemented for the functionalization of structurally related 3-aminochromones [150] and five-membered 2-aminomaleimides [151].

**Scheme 35:**
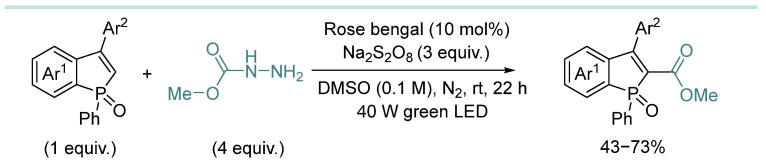
Radical alkoxycarbonylation of benzophosphoranes.

The C–H alkoxycarbonylation of benzophospholes with methyl carbazate offers a facile route to highly substituted phosphole derivatives (Scheme 35) [152]. Under metal-free photocatalytic conditions employing rose bengal, the electron-neutral, electron-donating and electron-withdrawing substituents were well tolerated.

**Scheme 36:**
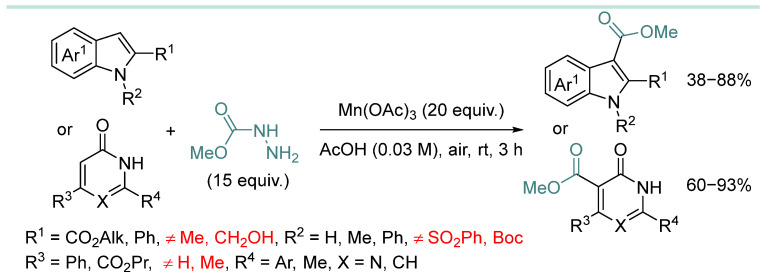
Radical alkoxycarbonylation of indoles, pyrimidinones and pyridinones.

Mn(OAc)_3_-promoted alkoxycarbonylation of indoles, pyrimidinones and pyridinones with methyl carbazate was recently reported (Scheme 36) [153]. The desired product was not obtained from indoles containing strong electron-acceptor groups, either on the benzene ring (e.g., NO_2_) or at indole nitrogen (e.g., R^2^ = SO_2_Ph).

**Scheme 37:**
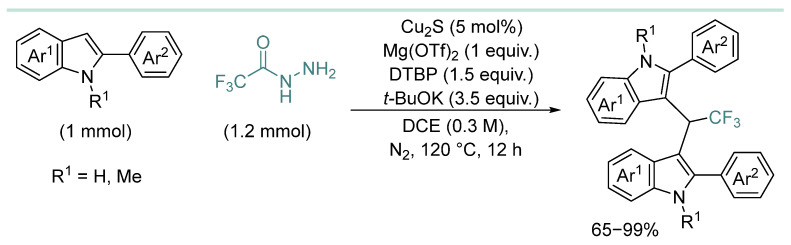
The synthesis of trifluoromethylated bis(indolyl)arylmethanes from 2-arylindoles and 2,2,2-trifluoroacetohydrazide.

The development of novel methods for introducing the trifluoromethyl (CF_3_) group is a high-priority objective in synthetic chemistry, owing to the group’s significant role in medicinal chemistry. Radical trifluoromethylation strategies are particularly valuable, as they facilitate the late-stage functionalization of pharmaceutically relevant compounds. The novel method describes the generation of trifluoroacetyl radical from trifluoroacetohydrazide with the copper catalysis [154]. The addition of trifluoroacetyl radical to the 3-unsubstituted indole leads to the trifluoromethylated bis(indolyl)arylmethanes (Scheme 37).

## 7. Miscellaneous Reactions

**Scheme 38:**
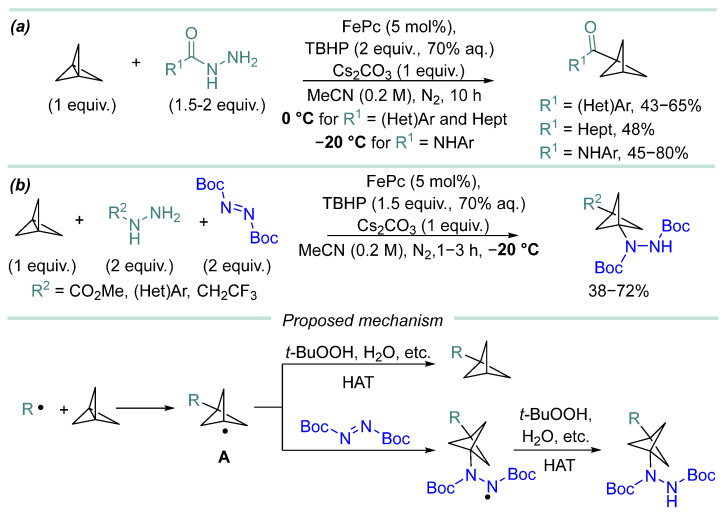
***(a)*** Hydroacylation of [1.1.1]propellanes ***(b)*** Functionalization of [1.1.1]propellanes with the C–O and C–N bond formation employing azodicarboxylate as C-radical scavenger.

[1.1.1]Propellanes have unique properties due to high strain energy. They readily accept radicals and convert to 1-substituted bicyclo[1.1.1]pentanes (Scheme 38a) [155]. The mechanism of radical addition to propellanes closely resembles that of radical addition to alkenes, described in Chapter 2.1. The chemical similarity is evidenced by the successful application of the FePc/TBHP oxidative system, which is effective for both substrates.

Later, multicomponent reaction between [1.1.1]propellanes, carbazates and azodicarboxylates under the similar conditions was developed (Scheme 38b) [156]. In this case, the intermediate carbon-centered-radical **A** is intercepted by azodicarboxylate to form corresponding hydrazine derivative before final HAT step.

**Scheme 39:**
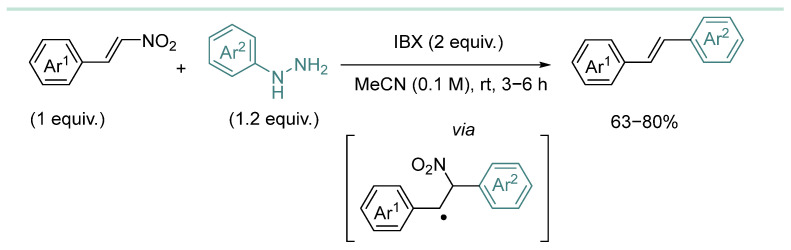
The synthesis of stilbenes via the addition of aryl radicals to β-nitrostyrenes.

The addition of carbon-centered radicals to β-nitrostyrenes leads to the formation of a new carbon–carbon bond, followed by elimination of a nitro group and, ultimately, leads to the synthesis of alkenes. Thus, reaction between β-nitrostyrenes and arylhydrazines in the presence of strong metal-free oxidant o-iodoxybenzoic acid (IBX) was shown as an effective method to produce stilbenes with excellent E-selectivity (Scheme 39) [157]. This method can also be extended to the synthesis of conjugated dienes from 1-nitro-1,3-dienes.

**Scheme 40:**
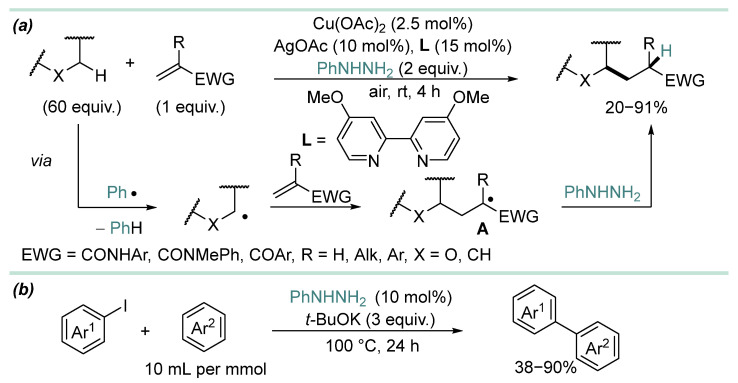
Radical reactions, initiated by phenyl hydrazine: ***(a)*** radical addition to Michael acceptors and ***(b)*** radical arylation of arenes by iodoarenes.

Although hydrazine-derived C-centered radicals usually undergo incorporation into products via radical addition processes, such radicals can also act as hydrogen atom abstractors for the generation of other carbon-centered radicals [158,159]. For example, phenylhydrazine can be effectively implemented as initiator for the difunctionalization of Michael acceptors with cyclic ethers and alkanes (Scheme 40a). In this reaction, phenyl radicals accept hydrogen atom from CH-substrate, whereas phenylhydrazine acts as a hydrogen atom donor for the intermediate radical adduct **A**.

Phenylhydrazine also serves as an efficient initiator for a radical-based direct arylation of arenes with aryl iodides (Scheme 40b) [160]. The mechanism involves base-activated phenylhydrazine transferring an electron to the aryl iodide, generating an aryl radical. This radical adds to the arene substrate in a chain process to form the biaryl product.

**Scheme 41:**
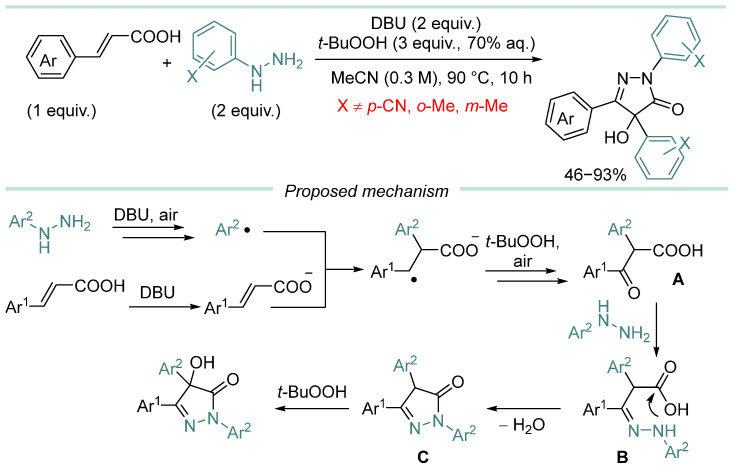
Oxidative cyclization of cinnamic acids with arylhydrazines.

A reaction between cinnamic acids and arylhydrazines was recently demonstrated as a novel path to 4-hydroxy-pyrazol-3-ones (Scheme 41) [161]. The reaction starts as a typical addition of aryl radical to vinylarenes, similar to those depicted in Scheme 1, followed by oxidation to arylketone **A** (Scheme 41). The condensation of arylketone **A** with the second equivalent of arylhydrazine gives hydrazone intermediate **B**, which undergoes intermolecular cyclization to pyrazol-3-one **C**. Being easily oxidizable, pyrazol-3-one **C** is readily converted to the final 4-hydroxypyrazol-3-one in the presence of *t*-BuOOH. Both electron withdrawing (F, CF_3_) and electron-donating substituents (MeO, Me) in the *para* position of the phenyl hydrazine are tolerated under the reaction condition except for *para*-CN substituent, which can be attributed to the slower oxidation of the electron-deficient arylhydrazine. *Ortho*- and *meta*- substituents failed to produce the desired product due to steric factors according to the authors [161]. No such limitations were observed for the substituents on the cinnamic acids. Moreover, reaction successfully proceeds with 3-(furan-2-yl)acrylic acid (71%) and 3-(thiophen-2-yl)acrylic acid (77%).

## 8. Conclusions

To sum up, hydrazine derivatives represent synthetically available, stable, convenient and atom-efficient C-centered radical precursors for C-C bond-forming syntheses under relatively mild conditions. The most developed areas in this field are alkene difunctionalizations (both electron-rich and -deficient, conjugated and unconjugated), cascade cyclization initiated by addition of hydrazine-derived C-centered radical to acrylamides and isonitriles, and (hetero)arene CH-functionalization. The following current limitations and challenges in this field can be highlighted:

- In most cases, hydrazine derivative is used in excess amounts, in some cases, C-radical acceptor is used in excess, but there are almost no effective examples of C-C coupling with 1:1 substrate/hydrazine ratio, which is a serious limitation when two complex and valuable reagents are to be coupled.
- Despite extensive development of the field, there is no established fundamental understanding of the rational oxidative system choice and prediction of reactivity for various hydrazine types. Apparently, arylhydrazines and alkylhydrazines without electron-withdrawing group are oxidized more easily compared to carbazates and acylhydrazines and, thus, oxidation by air/base systems (see Scheme 1d, Scheme 29, Scheme 32, Scheme 33 and Scheme 39; Table 4, entry 4; Table 5, entry 5; Table 7, entry 1; Table 8, entries 2, 5 and 6) becomes possible without the usage photoredox catalysts, peroxides or transition metal catalysts.
- The application of acylhydrazines and alkylhydrazines remains less explored compared to arylhydrazines and carbazates, despite the synthetic importance of methods for the introduction of alkyl and acyl substituents.
- There is a limited number of examples for radical hydrogen substitution in aliphatic functional groups (compared to heterocycle CH-functionalization) and alkyne functionalization (compared to alkene functionalization).

## Figures and Tables

**Table 1:** Reaction of *N*-(hetero)aryl-*N*-acrylamides with hydrazine-derived C-centered radicals to form oxindole derivatives. Red color represents substituents for which no target product was formed.

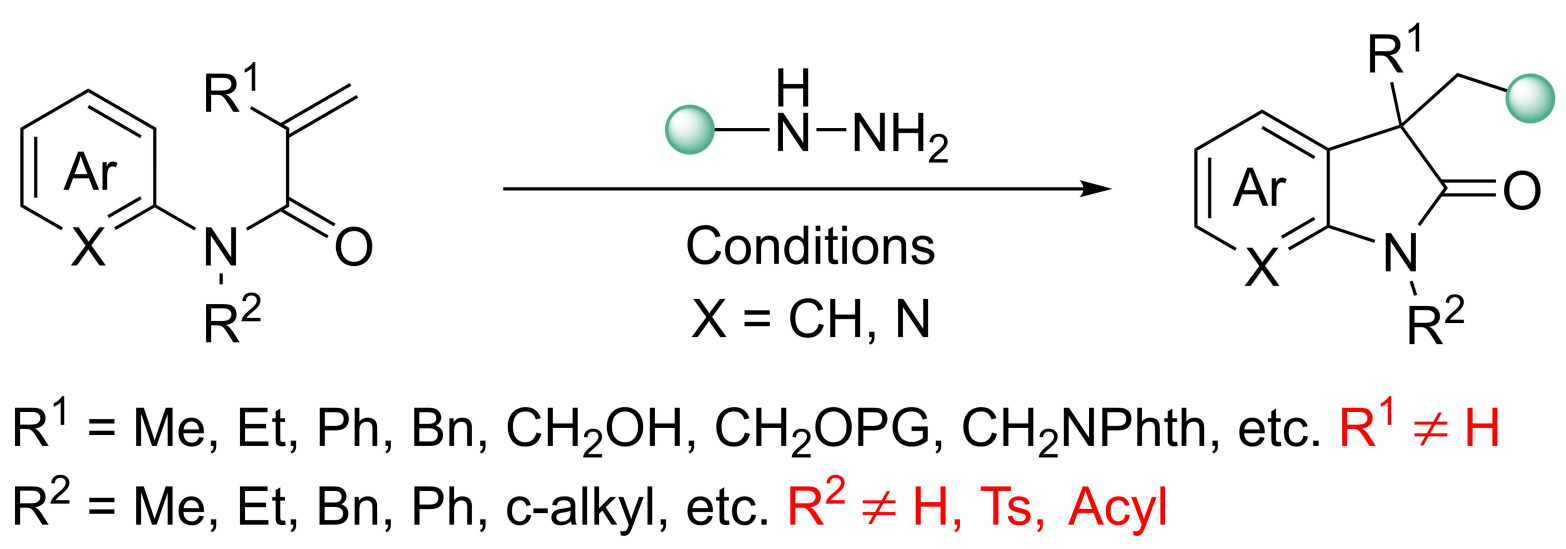			
№	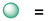	Conditions	Ref.
1	CO*t*-Alkyl, CO*i*-alkyl, COBn	HD (2 equiv.), **CuCO_3_** (10 mol%), **DTBP** (3 equiv.), DCE (0.1 M), 90 °C	[66]
2	CONHAr, CONH_2_, CONHAlk, CONHBn, CONHpropargyl.	HD (2 equiv.), **CuCO_3_** (1 mol%), **TBHP** (4 equiv), DCE/MeCN (1:1, 0.3 M), 70 °C, N_2_, 4 h.	[67]
3	CO_2_Me, CO_2_Et	HD (4 equiv.), **FeCl_2_·4H_2_O** (20 mol%), **TBHP** (6 equiv, 70% aq.), MeCN (0.125 M), 80 °C, 6 h	[68]
4	CO_2_n-Alk, CO_2_Ph	HD (4 equiv.), **FeCl_2_·4H_2_O** (10 mol %), 4-cyanopyridine (20 mol%), **TBHP** (5 equiv.), EtOAc (0.1 M), 80 °C, 4 h, air	[69]
5	CONHAr, CONHAlk. CONHBn, CO_2_Me, COPh, Ph	HD (2.5 equiv.), **Rose Bengal (5.0 mol%)**, **(NH_4_)_2_S_2_O_8_** (5 equiv.), DMSO/H_2_O (5/1, 0.06 M), 5 W blue LED (λmax = 480 nm), N_2_, rt, 48 h.	[70]
6	Ar	HD (1.5 equiv.), **Co(OAc)_2_** (10 mol%), NaOPiv (2 equiv.), EtOH (0.03 M), undivided cell, RVC(+)|Pt(−), **CCE 8 mA**, N_2_, rt, 12 h	[71]
7	CO_2_Alk	HD (2 equiv.), **FePc** (5 mol%), TBABF_4_ (1 equiv.), MeCN/DMSO/MeOH (2:1:1, 0.05 M), undivided cell, **CF(+)|Pt(−), 2.4 V**, 60 °C, N_2_, 15 h	[72]

**Table 2:** The synthesis of indole[2,1-α]isoquinolines from heterocyclic *N*-acrylamides.

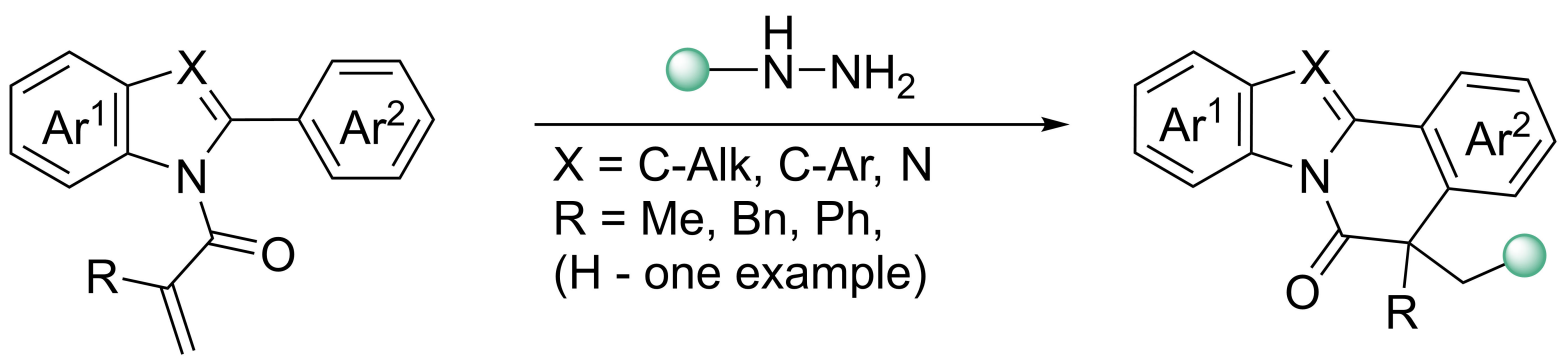			
№	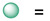	Conditions	Ref.
1	COAr	HD (8.0 equiv.), **CuI** (20 mol%), **TBHP** (8.0 equiv., 70% aq.), MeCN (0.1 M), 80 °C, 8 h.	[74]
2	CO_2_Me, CO_2_Bn, *t-*Bu	HD (5.0 equiv.), **FeCl_2_·4H_2_O** (20 mol%), **TBHP** (4.0 equiv., 70% aq.), MeNO_2_ (0.125 M), 80 °C, 6 h.	[75]
3	CO_2_Me, CO_2_Et, CO_2_t-Bu, CO_2_Bn	HD (6 equiv.), **FeCl_2_·4H_2_O** (20 mol%), **TBHP** (5 equiv.), DCE (0.1 M), 80 °C, 8 h.	[76]
4	CO_2_Me, CO_2_Et, *t-*Bu	HD (5.0 equiv.), **rose bengal** (5 mol%), **(NH_4_)_2_S_2_O_8_** (3.0 equiv.), DMSO (0.1 M), rt, 10 W blue LED, 15 h	[77]

**Table 3:** Addition of carbon-centered radicals derived from hydrazines to 2-isocyanobiphenyls. Red color represents substituents for which no target product was formed.

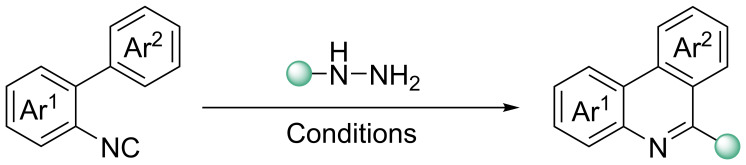			
№	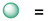	Conditions	Ref.
1	CO_2_Me, CO_2_Et, CO_2_n-Pr, CO_2_Ph, Ph, COMe	HD (4 equiv.), **FeCl_2_·4H_2_O** (0.02 mmol), phen·H_2_O (40 mol%), **TBHP** (5 equiv.), EtOAc (2 mL), 80 °C, air, 4 h	[87]
2	CO_2_Me, CO_2_Et	HD (2 equiv.), **Fe(acac)_2_** (5 mol%), **TBHP** (3 equiv., 70% aq.), PhF (0.1 M), 80 °C, N_2_, 12 h	[88]
3	CO_2_Me, CO_2_Et, ≠CO_2_Bn	HD (6 equiv.), **Bu_4_NI** (20 mol%), **TBHP** (5 equiv., 70% aq.), MeCN (0.15 M), 80 °C, 6 h.	[89]
4	Ar, Me, *t*-Bu, *c*-Hex, COPh, CO_2_Et	HD (3 equiv.), **eosin B** (5 mol%), K_2_CO_3_ (3 equiv.), DMSO (0.2 M), 5 W blue LED, rt, **air**, 18 h	[90]
5	Ar, *t*-Bu,	HD (3 equiv.), **2D-COF-1** (1 mol%), K_2_CO_3_ (3 equiv.), DMSO (0.1 M), 5 W Blue LED, rt, air, 20 h	[91]

**Table 4:** Free-radical functionalization of C-3 position imidazo[1,2-*a*]pyridines by hydrazine C-radical precursors.

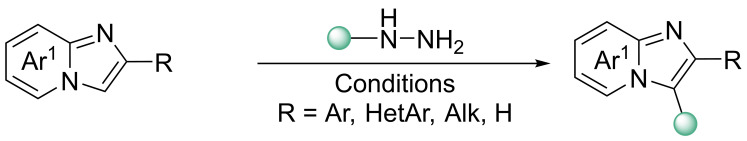			
№	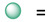	Conditions	Ref.
1	CO_2_Me, CO_2_Et	HD (2 equiv.), **FeCl_2_·4H_2_O** (20 mol%), **(NH_4_)_2_S_2_O_8_** (3 equiv.), DMSO (0.1 M), 55 °C, 6 h	[108]
2	CO_2_Me, CO_2_Et	HD (4 equiv.), **rose bengal** (5 mol%), **Na_2_S_2_O_8_** (3 equiv.), DMSO (0.08 M), blue LED, rt, 6−12 h.	[109]
3	CO_2_Me, CO_2_Et	HD (3 equiv.), **FePc** (5 mol%), NaHCO_3_ (3.0 equiv.), nBu_4_NHSO_4_ (1 equiv.) and MECN/HFIP (4.9/0.1, 0.04 M), Ar, CF(+)|Ni(−), **CCE 10 mA**, 6 h	[110]
4	Ar	HD (1.3 equiv.), DBU (4 equiv.), MeCN (0.6 M), rt, 4–12 h	[111]

**Table 5:** C–H functionalization of quinoxalin-2(*1H*)-ones with hydrazine derivatives.

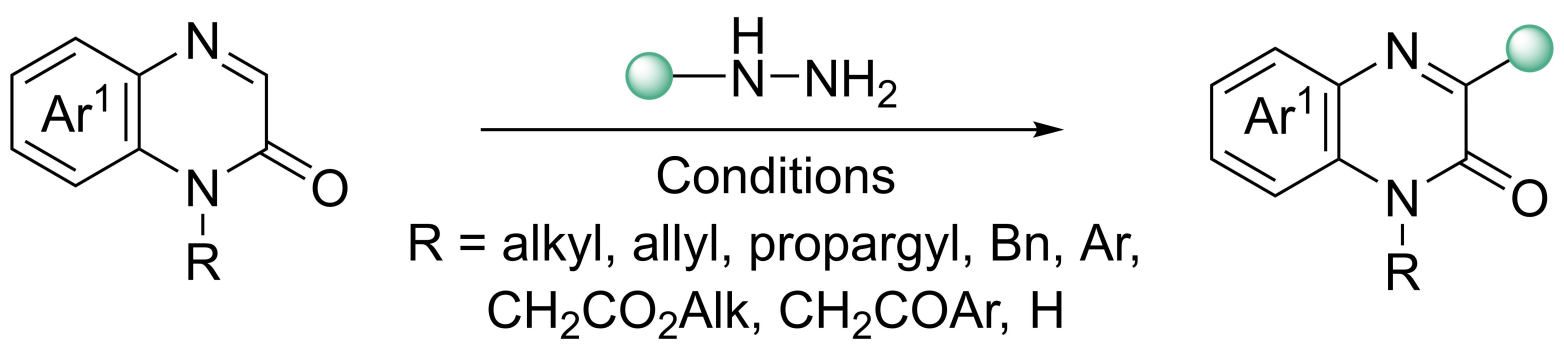			
№	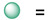	Conditions	Ref.
1	CO_2_Me, CO_2_Et, *t*-Bu, COAr	HD (1.5 equiv.), **K_2_S_2_O_8_** (3 equiv.), MeCN (0.2 M), 90 °C, 8–12 h	[115]
2	Ar	HD (1.5 equiv.), **K_2_S_2_O_8_** (3 equiv.), MeCN (0.1 M), 50 °C, 6–9 h	[116]
3	CONHAr	HD (2.5 equiv.), **CuI** (10 mol%), **BPO** (3 equiv.), DMSO (0.1 M), 100 °C, 18 h.	[117]
4	CO_2_Me, CO_2_Et, *t*-Bu	HD (4 equiv.), **rose bengal** (5 mol%), **Na_2_S_2_O_8_** (3 equiv.), DMSO (0.08 M), blue LED, rt, 6–12 h.	[109]
5	(Het)Ar, *c*-Hex, *i*-Pr,	HD (2 equiv.), **Eosin Y** (5 mol%), **KI** (1.5 equiv.), K_2_CO_3_ (3 equiv.), EtOH/H_2_O (2/1, 0.07 M), 12W blue LED, rt, 10 h	[118]
6	Ar	HD (2 equiv), **g-C_3_N_4_** (15 mg per 0.2 mmol), **NaI** (5 mol%), EtOH (0.07 M), air, rt, 8 W blue LED	[119]
7	Ar, alkyl	HD (3 equiv.), **2D-COF-1** (4 mg per 0.1 mmol), K_2_CO_3_ (3 equiv.), DMSO (0.06 M), 34 W blue LED, 24 h	[120]
8	Ph	HD (2.5 equiv), **PFC-943** (5 mg per 0.1 mmol), EtOH (0.1 M), O_2_ (1 atm.), 4 h, rt, 10 W white LED	[121]
9	Ar	HD (1.5 equiv.), DBU (3 equiv.), MeCN (0.2 M), **air**, rt, 8–12 h	[122]
10	Ar	HD (1.5 equiv), HCl (4.0 equiv, 37.5% aq.), MeCN (0.1 M), 25 W blue LED, air, rt, 10 h	[123]
11	Ar	HD (**1 equiv**.), MeCN (0.1 M), 4 W purple LED (390 nm), Ultrasound (44 kHz/30 W), rt, air, 4 h	[124]
12	Ar, *c*-Hex *c*-Pent, (CH_2_)_2_OH	HD (**1 equiv.**), MeCN (0.1 M), 400 nm LED, rt, 12 h	[125]
13	Ar, *c*-Hex	HD (2 equiv), Bu_4_NBF_4_ (1 equiv.), MeCN/H_2_O (9/1, 0.05 M), undivided cell, **CCE I = 10 mA**, C(+)|Pt(−), rt, 8 h	[103]
14	Ar, 2-quinolinyl	HD (3 equiv), **Quinuclidine** (20 mol%), LiClO_4_ (1 equiv.), NaHCO_3_ (1 equiv.), MeCN (0.06 M), undivided cell, **CCE I = 7 mA**, 50 °C, Pt(+)|Ni(−), 7 h, 6.09 F/mol	[126]

**Table 6:** The radical C3-functionalization of 2*H*-indazoles with hydrazine derivatives. Red color represents substituents for which no target product was formed.

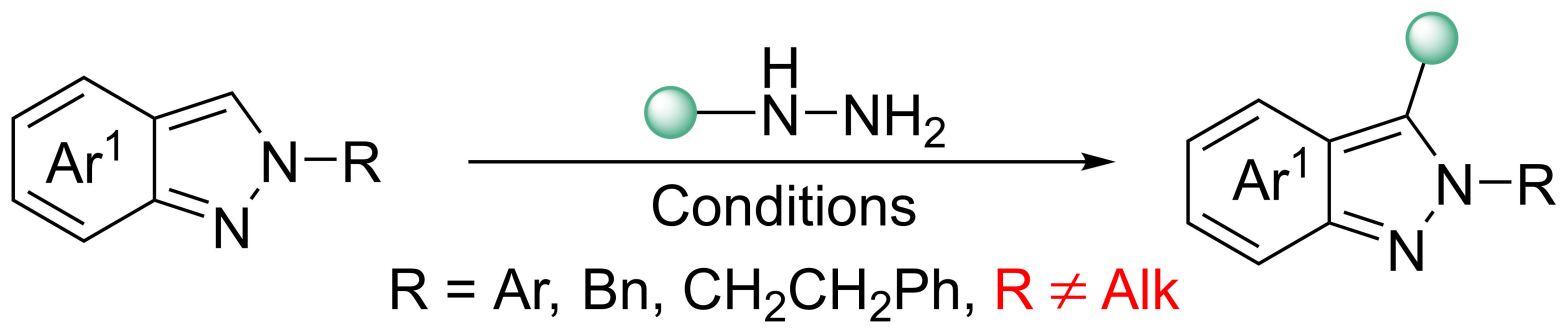			
№	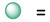	Conditions	Ref.
1	CO_2_Me, CO_2_Et	HD (2 equiv.), **TBHP** (4 equiv.), DCE (0.1 M), N_2_, rt, 8 h,	[130]
2	CO_2_Alk	HD (2 equiv.), Li_2_CO_3_ (1 equiv.), nBu_4_NOAc (0.1 M), MeCN/H_2_O (5/1, 0.6 M), undivided cell, C(+)|Ni(−), **CCE I = 6 mA**, rt, 10 h	[131]
3	Ar	HD (3 equiv.), **4CzIPN** (10 mol%), DIPEA (3 equiv.), MeCN, 7 W blue LED, **air**, rt, 24 h	[132]

**Table 7:** Reaction of quinones with hydrazine-derived radical species.

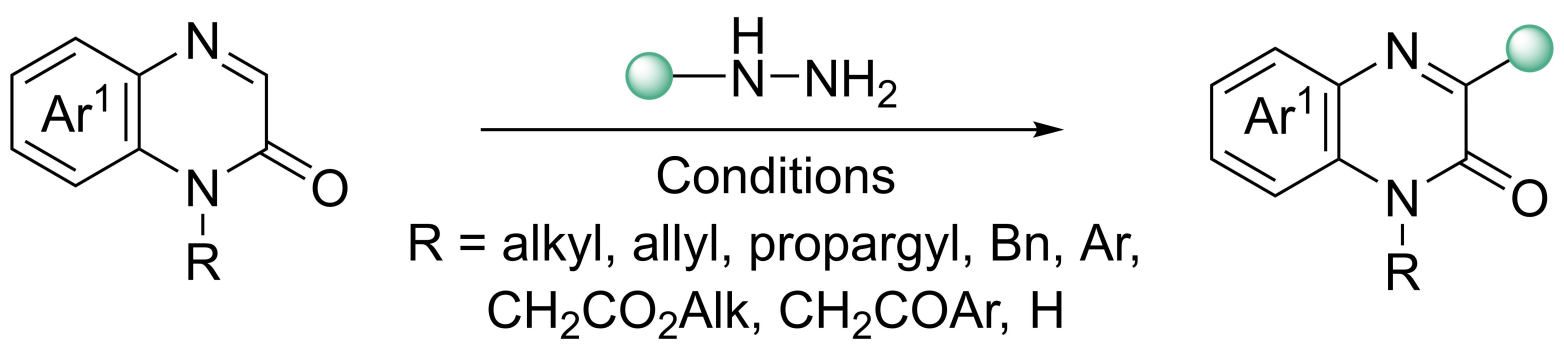			
№	R =	Conditions	Ref.
1	H	HD (1.2 equiv.), K_2_CO_3_ (3 equiv.), DMSO (0.07 M), rt, **air**, 12 h	[137]
2	NHR, NR_2_	HD (3 equiv.), Et_4_NOH (2 mol%), nBu_4_NBF_4_ (2 equiv.), MeCN (0.02 M), undivided cell, C(+)|C(−), **CCE 5 mA**, rt, 4 h	[138]
3	H, Br, Me, Ar, SAr, NR_2_	HD (1.2 equiv.), **I_2_** (0.3 equiv.), TFE (0.05 M), 40 °C, **air**, 16–20 h	[136]
4	H, Cl, Br, OH, NH_2_, NHAlk, Ph	HD (1.2 equiv.), **IBX** (2 equiv.), MeCN (0.05 M), 2.2–5 h	[139]
5	H	HD (1.2 equiv.), **IBX** (2 equiv.), MeCN (0.05 M), 3–5 h	[140]

**Table 8:** Reaction of anilines with hydrazine-derived radical species.

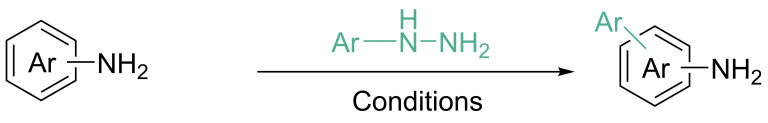		
№	Conditions	Ref.
1	aminoarene (20 equiv.), **MnO_2_** (5 equiv.), MeCN (0.2 M), rt, 2 h, **air**	[141]
2	aminoarene (20 equiv.), NaOH (1 M, 1 mL), 60–90 °C, 24 h, **air**	[142]
3	aminoarene (10 equiv.), NaOH (6 equiv.), MeCN/H_2_O (3/2, 0.2 M), 80 °C, 3 h, **air**	[146]
4	aminoarene (10 equiv.), CoPc (10 mol%), MeCN (0.1 M), 80 °C, 24 h, **air**	[145]
5	aminoarene (20 equiv.), K_2_CO_3_ (2 equiv.), DMSO (0.1 M), rt, **air**, 24 h	[143]
6	aminopyridine (20 equiv.), K_2_CO_3_ (3 equiv.), DMSO (0.1 M), rt, **air**, 24 h	[144]

## Data Availability

The original contributions presented in this study are included in the article. Further inquiries can be directed to the corresponding authors.

## Abbreviations

The following abbreviations are used in this manuscript:

4CzIPN	1,2,3,5-Tetrakis(carbazol-9-yl)-4,6-dicyanobenzene
BPO	benzoyl peroxide
CCE	constant current electrolysis
DCE	1,2-dichloroethane
DCM	dichloromethane
DTBP	di-*tert*-butylperoxide
DTBP	di-*tert*-butyl peroxide
FePc	iron phthalocyanine
HD	Hydrazine derivative
IBX	2-iodoxybenzoic acid
TBAI	tetra-n-butylammonium iodide
TBHP	*tert*-butylhydroperoxide
TBPB	*tert*-butylperoxybenzoate
TEMPO	(2,2,6,6-Tetramethylpiperidin-1-yl)oxyl
